# GD‐Net: An Integrated Multimodal Information Model Based on Deep Learning for Cancer Outcome Prediction and Informative Feature Selection

**DOI:** 10.1111/jcmm.70221

**Published:** 2024-12-04

**Authors:** Junqi Lin, Weizhen Deng, Junyu Wei, Jinyong Zheng, Kenan Chen, Hua Chai, Tao Zeng, Hui Tang

**Affiliations:** ^1^ School of Mathematics Foshan University Foshan China; ^2^ Guangzhou National Laboratory Guangzhou China; ^3^ GMU‐GIBH Joint School of Life Sciences, The Guangdong‐Hong Kong‐Macau Joint Laboratory for Cell Fate Regulation and Diseases, Guangzhou Laboratory Guangzhou Medical University Guangzhou China

**Keywords:** cancer prognosis, contrastive learning, deep learning, feature selection, multi‐modal integration

## Abstract

Multimodal information provides valuable resources for cancer prognosis and survival prediction. However, the computational integration of this heterogeneous data information poses significant challenges due to the complex interactions between molecules from different biological modalities and the limited sample size. Here, we introduce GD‐Net, a Graph Deep learning algorithm to enhance the accuracy of survival prediction with an average accuracy of 72% by early fusing of multimodal information, which includes an interpretable and lightweight XGBoost module to efficiently extract informative features. First, we applied GD‐Net to eight cancer datasets and achieved superior performance compared to benchmarking methods, with an average 7.9% higher C‐index value. The ablation experiments strongly supported that multi‐modal integration could significantly improve accuracy over the single‐modality model. In the deep case study of liver cancer, 319 differential genes, 15 differential miRNAs and 155 methylated differential genes based on the predicted risk subgroups are identified as the informative features, and then we have statistically and biologically validated the efficacy of these key molecules in internal and external test datasets. The comprehensive independent validations demonstrated that GD‐Net is accurate and competitive in predicting different cancer outcomes in real‐time, and it is an effective tool for identifying new multimodal prognosis biomarkers.

## 1 Introduction

Accurately evaluating cancer outcomes is crucial for selecting optimal treatment modalities, extending patients' survival time, and improving their quality of life. The efficacy of therapies is significantly hindered by the observed different outcomes among cancer patients receiving identical treatment. Accurate predictions of cancer outcomes are therefore essential to guide subsequent therapy decisions [1]. With the advancement of molecular sequencing techniques, the availability of multi‐modal datasets—such as mRNA‐seq, miRNA‐seq, methylation, clinical information and KEGG networks—has increased, offering valuable resources for assessing cancer outcomes and supporting clinical decision‐making [2].

Several methods have been proposed to predict cancer outcomes, such as the random survival forest (RSF) and similar methods, which often did not consider the patients' survival distribution [3, 4]. To improve predictions, dimensionality reduction techniques like PCA and kernel PCA have been integrated into Cox models for risk assessment [5, 6, 7, 8]. To address the computational challenges associated with high‐dimensional features, regularisation techniques such as the IPW‐lasso method [9] and an L1‐norm regularisation constant have been incorporated. Additionally, approaches like survival support vector machines (SSVM) and EXSA (based on decision trees) have enhanced performance by selecting appropriate kernel functions [10, 11]. However, traditional machine learning methods often struggle with high‐dimensional non‐linear data, failing to extract informative features.

Deep learning (DL) techniques have demonstrated significant advantages in handling non‐linear and high‐dimensional features [12, 13, 14, 15, 16, 17, 18, 19, 20, 21, 22]. DeepSurv, for instance, integrates deep neural networks with cancer survival tasks [23]. Chaudhary *et.al* used an autoencoder to compress high‐dimensional features, which were then applied in Cox regression for cancer survival analysis [24]. However, the separation of feature extraction and risk prediction limited convenience. To address this, an end‐to‐end deep learning network was introduced, combining cancer outcome evaluation with data recovery [25]. Building on this, Palmal et al. developed a graph convolutional network model that considers gene interactions during training [26]. Despite the superior performance of DL‐based methods in predicting cancer outcomes, their application remains constrained by the limited availability of training samples. To address the challenge of small sample sizes, researchers have developed transfer learning techniques [27], which augment model training with additional information. Recently, a self‐supervised learning strategy was developed for cancer research, enabling models to learn similarities and differences between samples [28, 29]. However, the performance of these models is still limited by batch effects from different datasets, which requires a strong assumption of data homogeneity.

In summary, the above methods have provided a good basis for predicting cancer outcomes, but challenges remain due to limited sample sizes and the rise of multimodal data. Integrating multimodal data for predicting cancer outcomes offers several advantages. (1) It enhances predictive accuracy by leveraging diverse data sources such as genomic, transcriptomic, and clinical information, providing a more comprehensive understanding of the disease. (2) It aids in the discovery of new biomarkers to improve early detection and prognosis. (3) It addresses tumour heterogeneity and provides a more robust and detailed analysis compared to single‐modality approaches, which is crucial for understanding resistance mechanisms and developing effective treatments.

Here, we propose an integrated multimodal information model (GD‐Net) based on Graph convolutional network and Deep self‐supervised contrastive learning network for precise cancer outcomes prediction and effective informative features extraction. First, GD‐Net implements an early integration scheme by generating a fusion matrix of the high‐dimensional multimodal input with data augmentation. This early‐fusion representation is next fed into a flexible ensemble of self‐supervised networks, combining deep learning and contrastive learning, to reconstruct multi‐omics meta‐features. These meta‐features are then used to evaluate cancer outcomes with an elastic‐net regularised Cox regression model. Additionally, GD‐Net incorporates an interpretable XGBoost module for informative feature selection. As shown by benchmark analysis, GD‐Net with multiple modalities as input is substantially more accurate than existing methods and outperforms its method with a single modality as input. Especially, in a case study of liver cancer, GD‐Net can effectively identify new biomarkers for cancer outcome prediction, whose statistical and biological significances have been widely validated in internal and external test datasets. GD‐Net also shows similar analysis efficiency in another case study of lung cancer. GD‐Net should be an effective tool for identifying new multimodal prognosis biomarkers.

## 2 Materials and Methods

### 2.1 The GD‐Net Model

**FIGURE 1:**
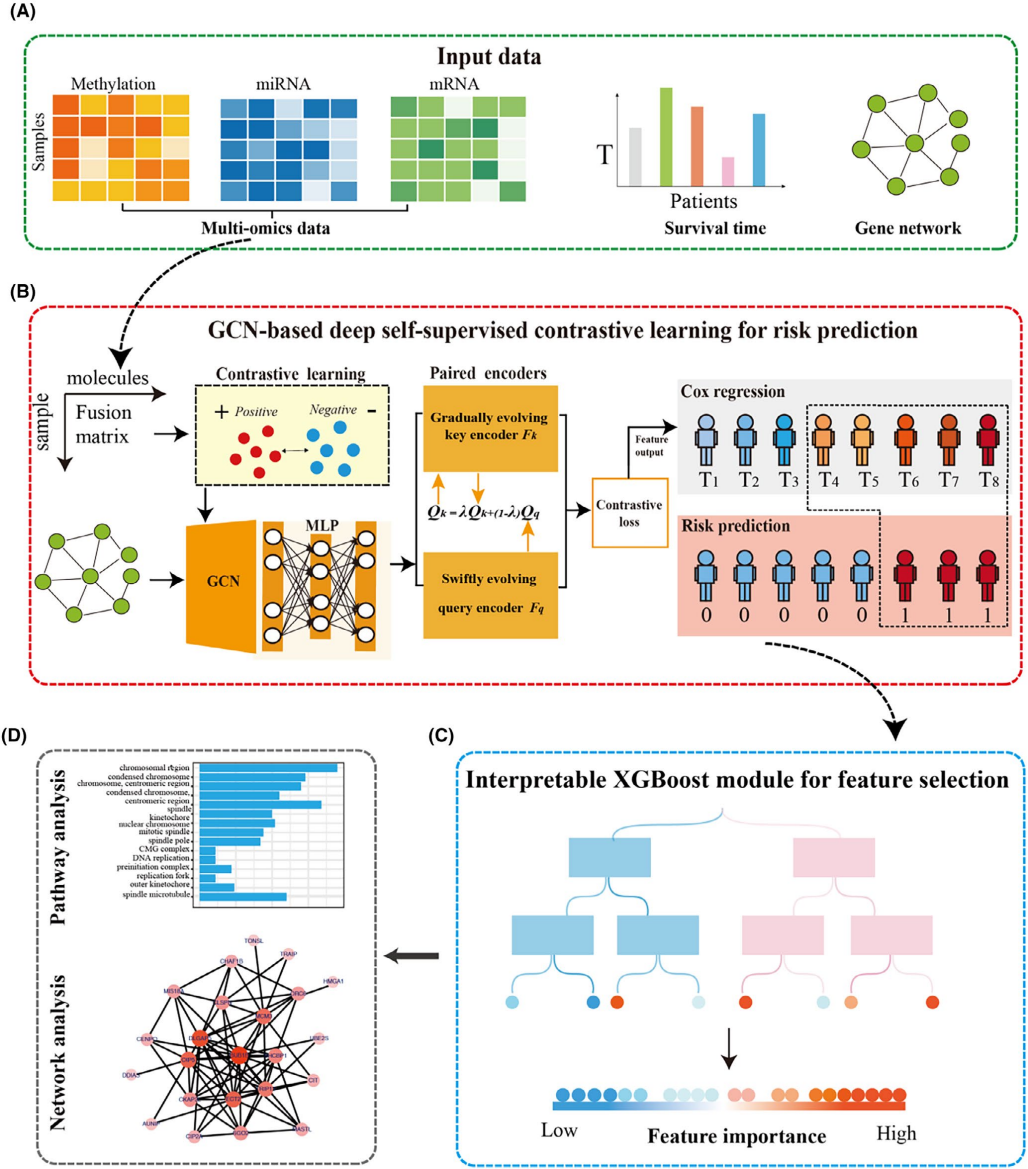
The workflow of GD‐Net. (A) The inputs for GD‐Net are a multimodal matrix (mRNA expression matrix, miRNA expression matrix and methylation matrix), a clinical survival information and a corresponding gene network from KEGG. (B) The GD‐Net framework includes contrastive learning for the generation of positive and negative pairs, the integration of GCN with a multilayer perceptron (MLP) layer for network learning and parameter passing, paired encoders combined with contrastive loss for the output of low‐dimensional representations, and a Cox‐Elastic Net (Cox‐EN) module for risk prediction. (C) The important component is an interpretable XGBoost module for informative feature selection. (D) The predicted risk subgroups and selected features by GD‐Net can be further investigated by diverse downstream analyses.

GD‐Net enhances the accuracy of cancer outcome (survival) prediction by incorporating multimodal data and prior knowledge of biological pathways through a graph convolution network (GCN) layer. The architecture of GD‐Net is illustrated in Figure 1. Initially, three types of multimodal data are fused using a stitching strategy. This fusion matrix, along with the KEGG network, is then processed by a flexible ensemble of self‐supervised networks that combine deep learning and contrastive learning. Subsequently, an elastic‐net regularised Cox model is employed for risk evaluation. Following the label prediction of patient risk, a lightweight XGBoost module is constructed to evaluate importance of multimodal features. Informative molecules both identified through our new XGBoost modelling and conventional differential expression analysis are multi‐omics meta‐features and considered to be potential cancer biomarkers. The details of GD‐Net construction are introduced step‐by‐step in below:

#### 2.1.1 Data Preparing

Assuming that we have three multi‐modal datasets ${\hat{X}}_{i}\epsilon {\mathbb{R}}^{m_{i}\times n^{\prime }\;}\left(i=1,2,3\right)$ from $n^{\prime }$ patients (each patients has $m_{i}$ number of features in each modality). $Y=\left(y_{1},y_{2},\ldots ,y_{n^{\prime }}\right)$ represents the clinical survival time for each patient. The KEGG gene connection pathway G for each cancer was used as the input network to the graph convolutional layer (GCN).

#### 2.1.2 Data Augmentation and Linear Stitching

To obtain more accurate information about the patients, we refer to Han's work [29]. During the model training process, data augmentation has been employed in GD‐Net to enhance the robustness of the deep neural network in acquiring additional informative features from cancer patients' data. After data augmentation, we obtained the new multimodal datasets $X_{i}\epsilon {\mathbb{R}}^{m_{i}\times n\;}\left(i=1,2,3\right)$ and $Y=\left(y_{1},y_{2},\ldots ,y_{n}\right)$ ($n^{\prime }<n$). Then, the fusion matrix X is constructed by linear stitching strategy:

(1) $$X=\text{rowbind}\;\left(X_{1},X_{2},X_{3}\right)$$

where rowbind represents matrix $X_{i}\epsilon {\mathbb{R}}^{m_{i}\times n}$ splicing by rows.

#### 2.1.3 GCN Combined With Contrastive Learning

By leveraging the inherent graph structure G, GCN layer can proficiently extract informative features through the fusion of genes from neighbouring nodes. The graph convolution formula is as follows:

(2) $$F\left(H^{\left(l\right)},A\right)=\sigma \left({\tilde{D}}^{{-}_{2}^{1}}\tilde{A}{\tilde{D}}^{{-}_{2}^{1}}\mathrm{HW}\right)$$

where A is the adjacency matrix of X, $\tilde{A}=A+I$, D is the degree matrix of X, W is the network parameter, $\sigma \left(∙\right)$ is the activation function, $F\left(H^{l},A\right)$ is the output of the GCN layer, $H^{l}$ represents the l th node feature matrix and H represents the input of the network G.

For extracting high‐quality multi‐omics information through contrastive learning, GD‐Net generates distorted samples as positive pairs and outputs distant representations of the features as negative pairs. $X=\left(x_{1},\ldots ,x_{m}\right)$ represents a list vector of multi‐omics features, where *m* denotes the number of features. In each batch, we set two similar features generated from the same sample as a positive pair, while considering the others as negative pairs. To distinguish between positive and negative pairs, GD‐Net utilises pairwise contrastive loss comparison in the following:

(3) $$L_{i,j}=-\log\frac{e^{\left(x_{i}∙\frac{x_{j}}{\tau }\right)}}{\sum_{k=1,k\neq i}^{2\mathrm{MN}}e^{\left(x_{i}∙\frac{x_{k}}{\tau }\right)}}$$

where *N* denotes the batch size and *M* represents the batch number. To alleviate over‐fitting in high‐dimensional feature scenarios, an L2‐regularised penalty module is incorporated as follows:

(4) $$L\left(x,x^{\prime }\right)=L_{i,j}+\gamma \sum_{i=1}^{k}F_{1\to i}{\left(x\right)}_{2}^{2}$$

where γ signifies the coefficient of the L2 norm regularisation part, *F*_*r*_ represents the node activity within the network, and *k* denotes the overall number of layers, encompassing input, output, and hidden layers.

Considering the heterogeneity of multimodal datasets, GD‐Net used a pair of encoders: a gradually evolving key encoder *f*_*k*_ and a swiftly evolving query encoder *f*_*q*_ to get the low‐dimensional representation of the multi‐omics features:

(5) $$\theta_{k}\leftarrow \lambda \theta_{k}+\left(1-\lambda \right)\theta_{q}$$

Let *θ*_*q*_ denote the parameters of the query encoder *f*_*q*_, and *θ*_*k*_ represents those of the key encoder *f*_*k*_. The query encoder update process utilised standard backpropagation, ensuring efficient parameter optimization. In contrast, the key encoder was updated using momentum‐based optimization, which enables consistent model updates along a stable trajectory.

#### 2.1.4 Cox‐EN Model for Risk Estimation

The evaluation of cancer outcomes was performed utilising the low‐dimensional representations extracted from the intermediate hidden layer of the contrastive loss network. These features were employed in the construction of elastic‐net‐regularised Cox proportional hazards (Cox‐EN) models. The Cox regression model for cancer outcome prediction is defined as follows:

(6) $$h\left(t|X_{i}\right)=h_{0}\left(t\right)\exp\left(\beta^{T}X\right)$$

where $h_{0}\left(t\right)$ represents the baseline hazard function of the disease outcomes change at time point t, and $\exp\left(\beta^{T}X\right)$ is risk change factors related to the coefficient vector β and the gene covariate vector X. Considering the coefficients $\beta_{i}$ of the gene i may be zero, the regularisation method elastic net $P\left(\beta_{j}\right)$ was added to the Cox model:

(7) $$l\left(\beta \right)=\arg\min_{\beta }\left\{l\left(\beta \right)+\lambda \sum_{j=1}^{p}P\left(\beta_{j}\right)\right\}$$

#### 2.1.5 Light‐Weighted XGBoost Algorithm for Informative Feature Selection

To identify informative features related to cancer outcomes, we trained an interpretable and lightweight model using the XGBoost algorithm with multimodal features. XGBoost is an advanced ensemble method that combines K decision trees (where X represents the molecule vectors and Y denotes the evaluated cancer outcome level by the deep neural network), making it an ideal choice for this study. Defining the training data comprises n patients with p features: $D=\left\{\left(x_{i},y_{i}\right)\right\}\;\left(\left|D\right|=n,x_{i}\in X,y_{i}\in Y\right)$, the XGBoost algorithm employs K decision trees to make predictions on cancer outcomes:

(8) $${\hat{y}}_{i}=\emptyset \left(x_{i}\right)=\sum_{k=1}^{K}f_{k}\left(x_{i}\right),f_{k}\in F$$

where $F=\left\{f\left(x\right)=w_{q\left(x\right)}\right\}\;\left(q:{\mathbb{R}}^{m}\to T,w\in {\mathbb{R}}^{T}\right)$ are the space of decision trees, q represents the decision tree structure, T represents the number of decision tree leaves, and $f_{k}$ is the decision tree structure q with the weight w. Similar to the Yang's study [15], the depth of decision trees was selected from 2 to 8.

#### 2.1.6 Settings of GD‐Net Training

For each input datasets X used in this study, we drew a training sample 80% of X, uniformly at random without replacement of each sample, and the remain 20% of X was used as test dataset. This split was chosen as a standard practice in machine learning to provide enough data for training while retaining a significant portion for testing and validation [12, 30]. This ratio is commonly used to balance the need for model learning and the ability to assess its performance on unseen data. To reduce the likelihood of bias and ensures that the model generalises well across different subsets of the data, we performed 5‐fold cross‐validation, which involves training and testing the model multiple times on different splits of the data. See Supporting Information for hyperparameter settings.

### 2.2 Model Evaluations and Methods for Comparisons

We evaluated the cancer outcome evaluation performance of GD‐Net by comparison with three typical methods (the PCA‐Cox model [31], eXGBC and the bridge regularisation‐based Cox method (Bridge‐Cox) [32]) and three DL‐based methods (Deep_surv [23], DCAP [15] and MTC [33]).

The performance of cancer outcome prediction is mainly evaluated by comparing C‐index (CI) and |log10(*p*)| values. The C‐index represents the proportion of correctly ordered pairs of patients based on Harrell's C statistics [10]:

(9) $$\mathrm{CI}=\frac{\sum_{i=1}^{n}\sum_{j\neq i}\delta \left[\left(r_{i}-r_{j}\right)\left(y_{i}-y_{j}\right)\geq 0\right]}{\sum_{i=1}^{n}\sum_{j\neq i}\text{comp}\left(i,j\right)}$$

where r is the patient's predicted risk, y is the patient's survival time, δ indicates the sample is uncensored or not. It should be noted that in the absence of censoring, $\text{comp}\left(i,j\right)=1$. The higher C‐index values suggest better cancer outcomes prediction. The value of |log10(*p*)| reflects the degree of survival differences among patient subgroups stratified by predicted risks, with higher values indicating greater statistical significance. Some other performance measurements are also used, such as AUC and ROC.

### 2.3 Informative Features Estimation

Differential expression analysis is employed to identify significant molecules with differential expressions among predicted risk sub‐groups. In this work, the differentially expressed molecules set $F_{\mathrm{DE}}$ was selected based on the criteria of |log2(fold change)| > 1.6 and corrected *p*‐value < 0.05. The top 200 molecules $F_{\mathrm{XGB}}$ ranked based on the importance values were selected as key features related to disease outcomes. The union of the set $F_{\mathrm{DE}}$ and $F_{\mathrm{XGB}}$ is the global informative molecules (IFMs), and the intersection of the set $F_{\mathrm{DE}}$ and $F_{\mathrm{XGB}}$ is the key informative molecules (key‐IFMs).

### 2.4 Statistics Analysis

The DESeq2 was used for differential expression analysis to detect the differentially expressed molecules set $F_{\mathrm{DE}}$ (https://github.com/thelovelab/DESeq2). The unpaired t‐test and principal coordinate analysis (PCoA) analysis were used to test and visualise the differences between the predicted subgroups. Permutational multivariate analysis of variance (PERMANOVA) was used to test the significance of PCoA results [34].

### 2.5 Enrichment Analysis

We carried out GO and KEGG functional enrichment analysis by the clusterProfiler package [35]. The key informative genes are linked and correlated by the combined functional couplings of protein–protein interactions of STRING (https://string‐db.org/). MicroRNAs which can regulate key DEGs were detected by ENCORI [36]. Survival R package (https://cran.r‐project.org/web/packages/survival/index.html) and Survminer R package (https://rpkgs.datanovia.com/survminer/) are used for survival analysis.

### 2.6 Datasets

In our research, we collected eight cancer datasets from the TCGA database (Table S1) using the R package “TCGA‐assembler 2” [37]. The experiment utilised three types of multi‐omics data, including mRNA, miRNA, and DNA methylation data. As DNA methylations provide information on millions of variables, we computed features at the gene level by taking the average of DNA methylation values across CpG sites that are located within each respective gene. In the stage of data preprocessing, we excluded gene features and samples with missing values exceeding 20%. Missing values in sample data were imputed using the median values of corresponding feature. mRNA and miRNA features were log2‐transformed for analysis. Finally, we used multiple omics features in eight cancer types, including 5849 mRNA, 1870 miRNA and 5721 methylation features which match the features of the KEGG network. The graph convolutional layer (GCN) was constructed utilising the information about the KEGG gene connection pathways. In addition, we also used the liver cancer RNA‐seq data from GSE54236 [38] for survival validation and assessed the The Cancer Genome Atlas (TCGA) database, encompassing 345 liver cancer samples with clearly defined cancer stages (e.g., Stage normal—IV) for informative genes validation.

## 3 Results

### 3.1 Overview of GD‐Net Method

GD‐Net is a hybrid and flexible computational framework to predict cancer outcome risk and estimate key molecules based on multimodal datasets (Figure 1). The first phase of GD‐Net is composed of early integration with linear stitching and various data augmentation techniques, including Gaussian noise, simulated dropout, data swap and data mask (Figure 1A). The second phase is composed of deep learning encoders, a type of GCN‐based deep self‐supervised contrastive learning approach. The deep learning encoders have 4 layers, the contrastive learning layer, the GCN layer, the paired encoders combined with contrastive loss layer, and the Cox‐EN module (Figure 1B). The contrastive learning layer effectively improves the feature representation by identifying similarities and differences between multimodal data samples. Then, the output, the fusion matrix and the KEGG network are fed into the GCN network. The output of the GCN layer is fed into paired encoders and a contrastive loss layer to output low dimensional features. The final Cox EN layer performs risk assessment by integrating the encoded information to predict patient outcomes. This modularized approach ensures robust and accurate modelling of multimodal data for cancer outcome prediction. Next, using light XGBoost approach, GD‐Net identifies the informative features of predicted survival subgroups (Figure 1C). This boosting approach enhances model interpretability by allowing easy identification of important features and their contributions to the prediction.

### 3.2 Benchmark Analysis of GD‐Net on Eight Typical Cancer Multi‐Omics Datasets

**FIGURE 2:**
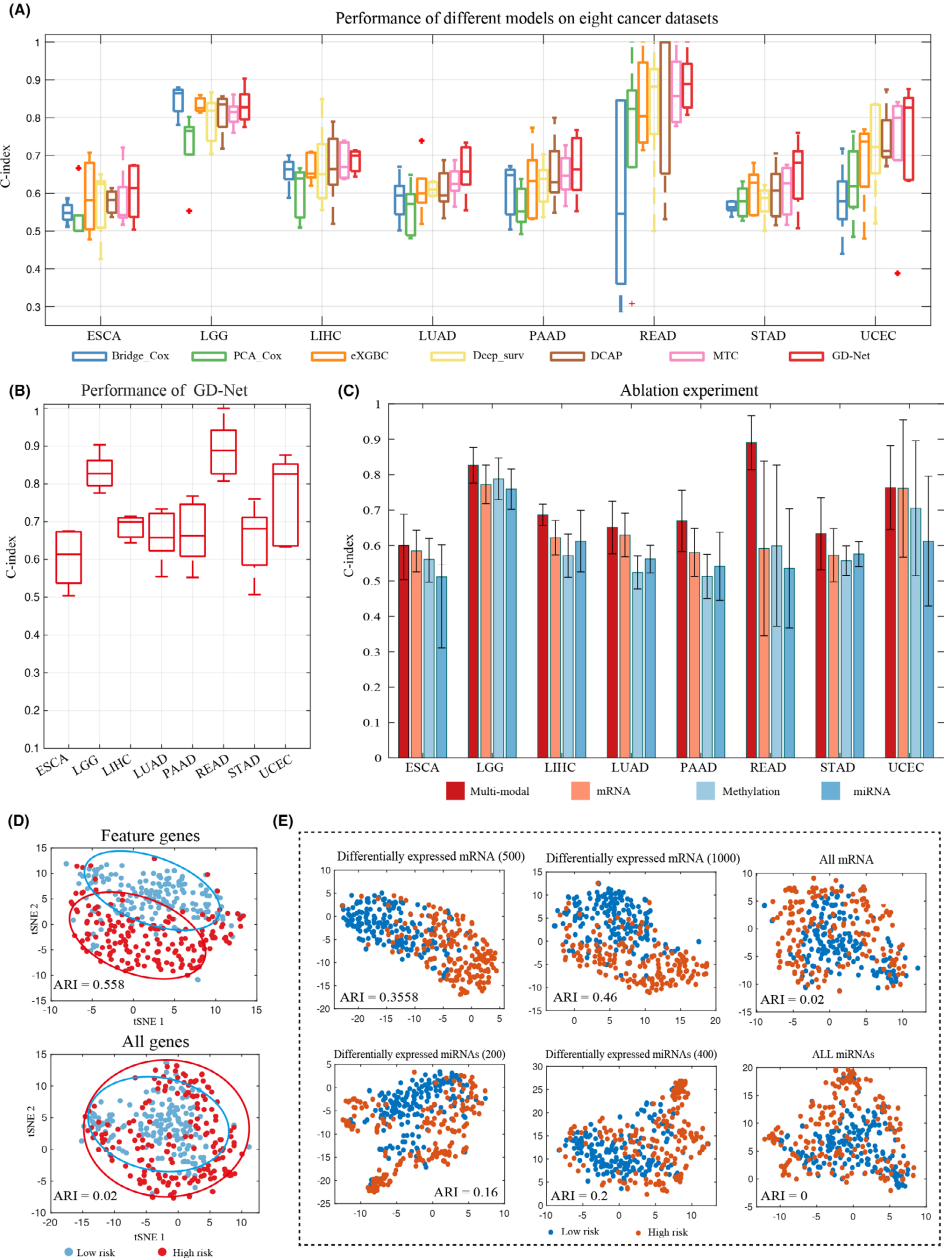
Benchmarking the prediction and feature selection on eight cancer datasets. (A) Performance comparison with benchmark models on eight cancer datasets. (B) The cancer outcome prediction performances of 5‐fold cross‐validation experiments obtained by GD‐Net on 8 cancers. (C) Benchmarking the performance of 5‐fold cross‐validation on single‐modality datasets as the ablation experiment. (D) The sample clustering is based on the feature molecules of the LIHC dataset selected by XGBoost from GD‐Net. By contrast, the baseline sample clustering is based on all molecules used as a shuffle group. (E) The sample clustering is based on the key molecules of the LIHC dataset selected by XGBoost. Again, the baseline sample clustering is based on all molecules used as a shuffle group.

We applied GD‐Net to analyse the multi‐omics data (mRNA‐seq, miRNA‐seq and DNA methylation) of 8 cancer datasets (Table S1). First, we compared the CI values obtained through 5‐fold cross‐validation using various methods including PCA‐Cox, eXGBC, Bridge‐Cox, Deep_surv, DCAP, MTC, and our proposed GD‐Net (see Methods). The CI obtained by GD‐Net ranged from 0.603 (ESCA) to 0.891 (READ), which achieves the highest average CI value (0.720) compared to other methods, indicating its superior performance (Table 1, Figure 2A). Compared to the other DL‐based methods, GD‐Net outperformed all cancer datasets. These findings suggest that the implementation of GD‐Net has improved the deep learning framework, which resulted in a significant improvement on average in CI evaluation (Figure 2B). In addition, we compared the AUC values obtained through 5‐fold cross‐validation across various methods. The AUC values and ROC curves demonstrated that GD‐Net consistently achieved comparable or superior performance relative to other models (Figure S1). The above results suggested that GD‐Net exhibited remarkable improvements compared to the other methods of cancer outcome prediction.

**TABLE 1:** The CI evaluation summary of 5‐fold CV for GD‐net compared to different methods on 8 cancer datasets.

	Bridge‐Cox	PCA‐Cox	eXGBC	Deep_surv	DCAP	MTC	GD‐Net
ESCA	0.549 (±0.025)	0.533 (±0.067)	0.59 (±0.088)	0.572 (±0.083)	0.577 (±0.029)	0.581 (±0.073)	0.603 (±0.068)
LGG	0.845 (±0.037)	0.728 (±0.089)	0.833 (±0.018)	0.794 (±0.059)	0.810 (±0.051)	0.811 (±0.033)	0.831 (±0.043)
LIHC	0.656 (±0.038)	0.602 (±0.063)	0.668 (±0.034)	0.669 (±0.101)	0.672 (±0.090)	0.684 (±0.044)	0.686 (±0.027)
LUAD	0.585 (±0.055)	0.555 (±0.062)	0.611 (±0.071)	0.611 (±0.017)	0.610 (±0.052)	0.629 (±0.040)	0.662 (±0.063)
PAAD	0.608 (±0.066)	0.564 (±0.051)	0.626 (±0.089)	0.623 (±0.056)	0.656 (±0.084)	0.649 (±0.054)	0.670 (±0.077)
READ	0.582 (±0.231)	0.750 (±0.233)	0.837 (±0.110)	0.826 (±0.171)	0.845 (±0197)	0.872 (±0.084)	0.891 (±0.068)
STAD	0.563 (±0.015)	0.577 (±0.039)	0.606 (±0.055)	0.576 (±0.041)	0.602 (±0.067)	0.607 (±0.063)	0.651 (±0.086)
UCEC	0.580 (±0.089)	0.630 (±0.094)	0.681 (±0.107)	0.722 (±0.116)	0.746 (±0.071)	0.729 (±0.172)	0.763 (±0.106)
Ave	0.621	0.617	0.682	0.674	0.690	0.695	0.720
*t*‐test *	1.45E−2	5.00E−6	2.57E−3	1.10E−4	6.56E−4	4.75E−4	—

* The *t*‐tests *p*‐values of each benchmark method by comparisons with GD‐Net.

### 3.3 Efficiency of GD‐Net on Multi‐Modality Analysis

As the ablation experiment, we calculated the contribution of each type/modality of omics data for cancer outcome prediction. When using each single‐modality of multi‐omics data as input of GD‐Net (Figure 2C), the mRNA data achieved the highest average CI value of 0.64 in most cancers, and the miRNA got the lowest one of 0.589 due to the bad performance in READ (CI = 0.535), and the methylation data (average CI = 0.603) performed better than miRNA and is close to mRNA. Although mRNA has the most amounts of important hidden features towards survival prediction, using multi‐modal data for cancer outcome prediction can usually significantly improve the CI value over the one only using mRNA data.

Then, as a representative example, the feature molecules of liver cancer for each type of omics data were identified (Table S2), and two predicted subgroups (i.e., 177 high‐risk and 180 low‐risk samples) can be well clustered into two discriminative groups (ARI = 0.56) based on key feature molecules (Figures 2D and S2). We also detect the significantly differentially expressed molecules of the two predicted subgroups (|log2(fold change)| > 1.6 and adjusted *p*‐value < 0.05), which can well differentiate the predicted risk groups (Figure 2E).

In addition, we evaluated the hardware requirements for real‐time implementation of GN‐net on the GPU platform, showing promising performance in processing speed and resource efficiency. The linear correlation between sample numbers and computational time indeed indicates that the GN‐net model can handle increasing data volumes without a significant rise in computational demands, making it suitable for large‐scale applications (Figure S3).

The above results all supported that GD‐Net can efficiently fuse information from multi‐modal data and achieve higher accuracy and robustness in predicting cancer outcomes.

### 3.4 Identification of Informative Features for the High‐Risk Group of Liver Cancer

**FIGURE 3:**
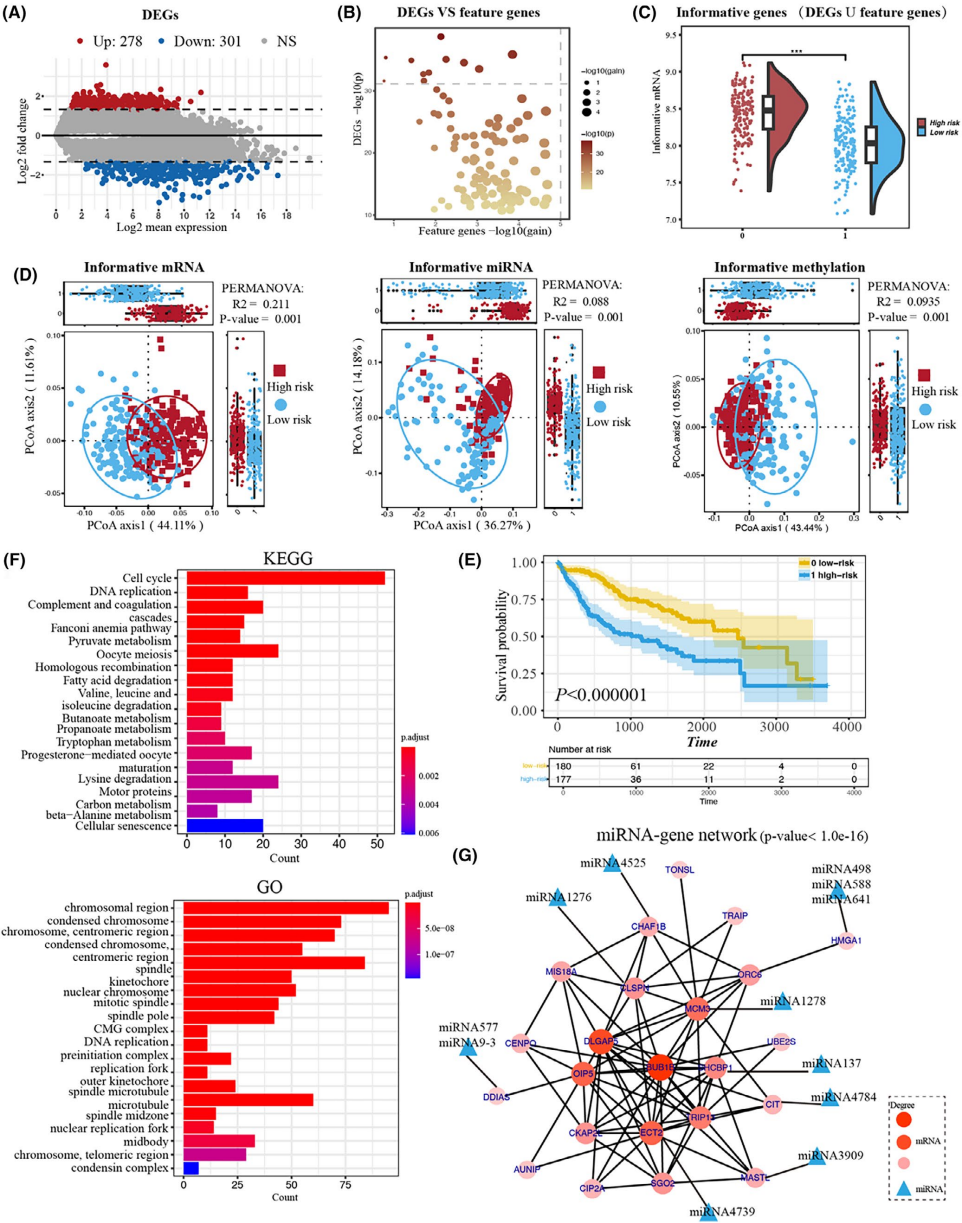
The case study on liver cancer. (A) The differentially expressed genes for risk groups identified by DESeq2 in liver cancer. The blue nodes represent down‐regulated risk genes, and the red nodes indicate up‐regulated risk ones (|log2(fold change)| > 1.6 and adjusted *p*‐values < 0.05). (B) The correlation plot between feature genes selected by GD‐Net and DEGs. (C) The half violin plot shows the average expression of informative genes between two risk groups. (D) Quantification of the expression profile differences of informative genes between two risk groups. (E) The Kaplan–Meier survival curves show the clinical relevance of the predicted two risk groups. The statistical *p*‐values were determined by the two‐tailed log‐rank sum test. (F) The enrichment bar plot shows the significantly enriched pathways of informative genes (FDR < 0.05). (G) The highly connected network consists mainly of 23 key informative genes and 12 relevant miRNAs. The 12 miRNAs have 9 target genes according to the ENCORI database. The size and graduated colour of the node indicate the network degree of each gene. And the PPI enrichment *p*‐value of genes is less than 1E−16.

To illustrate the identification and efficiency of GD‐net for key informative features associated with patient survival, we carried out a deep case study for liver cancer (i.e., LIHC dataset). Based on the identified risk subgroups by GD‐Net, 490 informative features (319 genes, 15 miRNAs and 155 methylated genes) were selected. Meanwhile, 579 DEGs (301 down‐regulated risk genes and 278 up‐regulated risk ones) with adjusted *p*‐value < 0.05 and |log2(fold change)| > 1.6 were selected by DESeq2 (Figures 3A and S4). There are 103 genes as key informative genes and 735 genes as global informative genes. In fact, many DEGs are positively correlated with predicted rank values (thought as functional gain) from GD‐Net (Figure 3B), thus GD‐Net is able to identify and select consensus informative genes. These informative genes showed the significant expression difference between two risk subgroups (Figure 3C). Furthermore, the PCoA analysis on these multi‐omics informative features with significance test by permutational multivariate analysis of variance (PERMANOVA) displayed a clear tendency of separation between the two risk subgroups of samples (Figure 3D). Particularly, on test samples from independent cohort, the survival analysis on predicted risk subgroups supported the reasonability of GD‐Net prediction (Figure 3E).

All informative genes can not only discriminate risk groups but also reveal insightful biological functions. By functional enrichment analysis, many KEGG pathways (including cell cycle, complement and coagulation and fatty acid degradations) and GO terms (including chromosomal region, spindle pole, centromeric region) relevant to abnormal cell functions in liver cancer were found (Figure 3F). Particularly, several key genes, such as *SLC27A5*, *IGF2*, *EFNA3*, *DCAF4L2*, and *SPP1*, have been demonstrated to be associated with liver cancer (Table 2). In vitro assays, *SLC27A5* holds promise as a potential prognostic marker for liver cancer [39] and *IGF2* has been linked to unfavourable clinical outcomes [40]. *DCAF4L2* has been shown to promote the progression of liver cancer [41], while *EFNA3* and *SPP1* have been treated as prognostic targets for survival in hepatocellular carcinoma patients [42, 43]. Notably, *INMT, ANO1* and *GCNT3* have recently been implicated in other types of cancer [44, 45, 46], which could potentially impact the outcomes of liver cancer. In addition, we applied the trained model on another independent liver cancer datasets to validate these genes by survival analysis (Figure S5) and found a significant difference of these genes between predicted high and low risk groups.

**TABLE 2:** The identified key informative genes related to liver cancer outcomes.

Gene	|logFC|	AveExp	*p*	Importance
SPP1	2.188	11.306	5.92E−08	1.69E−02
ACKR1	2.044	4.433	1.34E−14	1.02E−02
SLC27A5	2.011	12.402	5.31E−16	7.45E−04
GCNT3	2.009	5.363	6.17E−11	1.21E−03
INMT	2.005	6.809	1.02E−21	8.20E−03
GAGE2D	1.926	1.555	3.00E−08	2.85E−03
IGF2	1.709	11.682	2.94E−04	2.36E−03
DCAF4L2	1.353	3.401	6.96E−03	1.30E−03
ANO1	1.330	9.577	7.35E−08	1.12E−03
ITIH3	1.281	14.772	2.38E−08	6.86E−03
EFNA3	1.010	6.292	5.28E−12	4.03E−03

Abbreviations: |logFC|: the |log2FoldChange| values obtained in differential expression analysis; AveExp: Average expression in LIHC dataset; Importance: The feature importance values computed by XGBoost from GD‐Net; *p*‐value: the adjusted *p*‐value between the different risk groups identified by GD‐Net.

Furthermore, the first principal axis (PCoA1) analysis explains lower variance (42.78%) in the miRNA data compared to that of PCoA1 in the mRNA and methylation data (Figure 3D). This result is in line with the above findings that mRNA data would play the most important role in cancer outcomes prediction while miRNA made the least contribution. However, these key miRNAs are closely linked to the function and the evolution of their targeted key genes. As an illustrative instance, we constructed gene–gene network of key informative genes based on STRING (*p*‐value < 10E−16, Figure 3G). Strikingly, 12 key miRNAs are correlated with 8 key informative genes in this network. miRNA‐1276 has been used to explore the therapy strategy of hepatocellular carcinoma [47]. According to the enrichment analysis (Table S3), this network is significantly enriched with adrenomedullin receptor activity relevant to liver cancer [48]. We also found that the hub upregulated genes in this network—*MCM3, BUB1B, DLGAP5, DIP5, ECT2*—have been linked to liver cancer according to wide literature reports. For example, the upregulated *DLGAP5* significantly promotes the proliferation and invasion of HCC cells [49]. *MCM3* expression was associated with increased tumour invasion in HCC tissues [50]. *BUB1B* promotes hepatocellular carcinoma progression via activation of the mTORC1 signalling pathway [51]. Their expression levels were then cross‐referenced with data from the TCGA cohort, which included 345 liver cancer patients with five different grades (normal, I to IV). Our analysis revealed that certain genes, including *MCM3, BUB1B, DLGAP5* and *ECT2*, exhibited a noticeable increase in expression form stages I to stage III (Figure S6B). The findings support the above result that these key genes may serve as key‐warning indicators in the early stage of hepatocellular carcinoma development, which appear to be a promising diagnostic biomarker for liver cancer.

We also evaluated the average DNA methylation levels of informative methylated genes between the two risk groups and the average expression levels of corresponding genes that were significantly differentially expressed in RNA‐seq data (*p*‐value < 0.05). Our analysis showed that most genes exhibited lower methylation levels in the high‐risk group compared to the low‐risk group (Figure S7A). This suggests an inverse relationship between DNA methylation and gene expression in liver cancer prognosis, where reduced methylation is linked to higher risk and likely contributes to gene expression changes associated with poor outcomes. For example, the previously mentioned informative gene *ECT2* exhibits lower DNA methylation levels and higher expression levels in high‐risk groups. Overexpression of *ECT2* has been reported to be associated with cancer, and the DNA methylation status in promoter regions plays a key role in regulating its expression during cancer progression [52, 53] (Figure S7B). Similarly, expression of *SFN* is controlled epigenetically by DNA demethylation, and its overexpression is significantly correlated with poorer outcome [54]. Both *SFN* and *S100P* have been shown to be involved in cancer progression [54, 55, 56] (Figure S7B). These results further validate that the multi‐modal informative features selected by GD‐Net are crucial in the early stages of liver cancer, offering a more comprehensive understanding of the molecular mechanisms driving cancer development.

In conclusion, GD‐Net can accurately predict cancer outcomes and could integrate the multi‐regulatory network based on multimodal data. All relevant informative genes, microRNAs and methylated genes provide a basis for the multi‐gene diagnosis of cancer.

## 4 Discussion

Multi‐omics data are valuable resources for cancer prognosis and survival prediction; however, there are many challenges to integrate such multimodal data. Here, we present a novel and general computational framework, named GD‐Net, specifically for risk prediction and feature selection. GD‐Net can process multiple types of omics datasets and relevant biological network knowledge by a combination of GCN‐based self‐supervised deep neural network and interpretable XGBoost module, where some unique computational characteristics of GD‐Net contribute to its satisfied performance compared to existing methods:

1. GD‐Net uses the multimodal data as the feature matrix and the priori network of KEGG as the network structure, which are input to the following GCN. Such input design considers the biological gene network information, which makes the prediction more robust due to the consideration of the interplay between features from different modalities.
2. GD‐Net uses a GCN framework incorporated with contrastive learning that increases the accuracy of the final model, by maximising the similarity between related patients and minimising the similarity between unrelated patients. Contrastive learning improves the quality of feature representations and helps models generalise better to new, unseen data. Then, the hidden layer features obtained by the contrastive loss layer are subsequently fed into a unified Cox‐Elastic Net (Cox‐EN) model for risk prediction. The Cox‐EN model combines the strengths of Cox proportional hazards modelling with elastic net regularisation. It effectively handles high‐dimensional data, prevents over‐fitting by applying both L1 and L2 regularisation, and selects relevant features, thereby enhancing the model's predictive performance and interpretability in survival analysis.
3. GD‐Net uses an interpretable module based on the XGBoost algorithm to realise feature selection, which increases the model interpretability.

In this study, we demonstrated the value of GD‐Net in integrating multimodal data. GD‐Net performs significantly better overall than all other methods at predicting patient survival. One major reason is that other methods does not consider gene regulatory network when performing integration, and they only rely on the patterns from multiple types of genomics data, which would be usually mislead by limited data from a small number of samples. Especially, the ablation analysis utilising diverse omics data, revealed that mRNA exhibited the optimal performance, followed by methylation and miRNA in the second and third positions, respectively. These results provide new evidence for the complementary information from different levels of biological system in diseases, indicating the importance of multimodal information integration again.

During a deep study on liver cancer, we have identified 360 informative features that exhibit a high degree of association with liver cancer prognosis. There are more than 80% overlaps between informative features and DEGs (Figure S2); thus, the key informative genes were extracted for downstream analysis, which have high sensitivity and good performance for clustering of risk groups of liver cancer samples. In addition, we have also performed another similar case study on Lung adenocarcinoma, including identifying informative features, PCoA analysis, functional enrichment analysis and survival analysis (Figure S8). Various statistical and biological downstream analyses revealed the relevance of these key informative features for lung cancer, such as biomarker identification, biological function analysis and survival analysis.

Although GD‐Net has been demonstrated to be accurate and robust in predicting cancer outcomes, there are still some questions that warrant further discussion. Diverse imaging modalities utilised in cancer diagnosis and prognosis can furnish valuable insights into the progression of tumours. The incorporation of image and omics data should hold the potential to augment the predictive performance [57, 58]. In future research, it is necessary to improve the accuracy of cancer outcome prediction by constructing a multi‐task deep learning framework based on the integration of additional multi‐modal information from CT, MRI images and single cell datasets combined with spatial transcriptomes.

## Author Contributions

**Hui Tang:** methodology (lead), writing – original draft (lead). **Junqi Lin:** methodology (supporting), resources (lead), software (lead), validation (lead). **Weizhen Deng:** data curation (lead). **Junyu Wei:** investigation (lead). **Jinyong Zheng:** investigation (equal). **Kenan Chen:** validation (supporting). **Hua Chai:** validation (supporting). **Tao Zeng:** writing – original draft (equal), writing – review and editing (equal).

## Conflicts of Interest

The authors declare no conflicts of interest.

## Supporting information


Appendix S1.

## Data Availability

The method codes are available at https://github.com/JackiLin/GD‐Net.
