# Supplementary material for: GD‐Net: An Integrated Multimodal Information Model Based on Deep Learning for Cancer Outcome Prediction and Informative Feature Selection
Source: J Cell Mol Med. 2024 Dec 4;28(23):e70221. doi: 10.1111/jcmm.70221 (PMC11615516; doi:10.1111/jcmm.70221)
Supplement: Supplementary file 1 — Appendix S1. [file JCMM-28-e70221-s001.docx]

**Supplementary information for**

**GD-Net：A integrated multi-modal information model based on deep learning for cancer outcomes prediction and informative feature selection**

Junqi Lin^1^, Weizhen Deng^1^, Junyu Wei^1^, Jinyong Zheng^1^, Kenan Chen^1^, Hua Chai^1^, Tao Zeng^2,3^ *, Hui Tang^1^*

^1^ School of Mathematics, Foshan University, Foshan 528000, China.

^2^ Guangzhou Laboratory, Guangzhou, China.

^3^ GMU-GIBH Joint School of Life Sciences, The Guangdong-Hong Kong-Macau Joint Laboratory for Cell Fate Regulation and Diseases, Guangzhou Laboratory, Guangzhou Medical University, Guangzhou, China.

*Corresponding author: Tao Zeng, Email: [zeng_tao@gzlab.ac.cn](mailto:zeng_tao@gzlab.ac.cn); Hui Tang Email: [tanghui@fosu.edu.cn](mailto:tanghui@fosu.edu.cn)

**NOTE:** Settings of Hyper-Parameters

**Figure S1** Performance comparison with other models on eight cancer datasets based on AUC values.

**Figure S2** GD-Net model accurately identifies informative genes.

**Figure S3** Time cost of GD-Net on eight cancer datasets.

**Figure S4** Case study on liver cancer.

**Figure S5** The prediction results of additional datasets GSE54236.

**Figure S6** Additional dataset validation of informative genes.

**Figure S7** Association analysis between informative methylated genes and relevant genes.

**Figure S8** Case study on Lung adenocarcinoma (LUAD).

**Table S1** The statistical information about the cancer experimental datasets

**Table S2** The informative features select by GD-Net

**Table S3** Enrichment analysis of Figure 3G

**NOTE: Settings of Hyper-Parameters**

The parameter list for this study is as follows: Hidden layer 1 consists of 1024 nodes, while hidden layer 2 has 128 nodes. The node number in middle hidden layer was chosen from the set [10, 20, 50]. The learning rate (LR) was chosen from the range [1e-2, 1e-3, 1e-4, 1e-5], and the maximum epoch for training was set 200. The momentum coefficient was set 0.99. The parameters were selected based on the 5-fold results in the experiments.


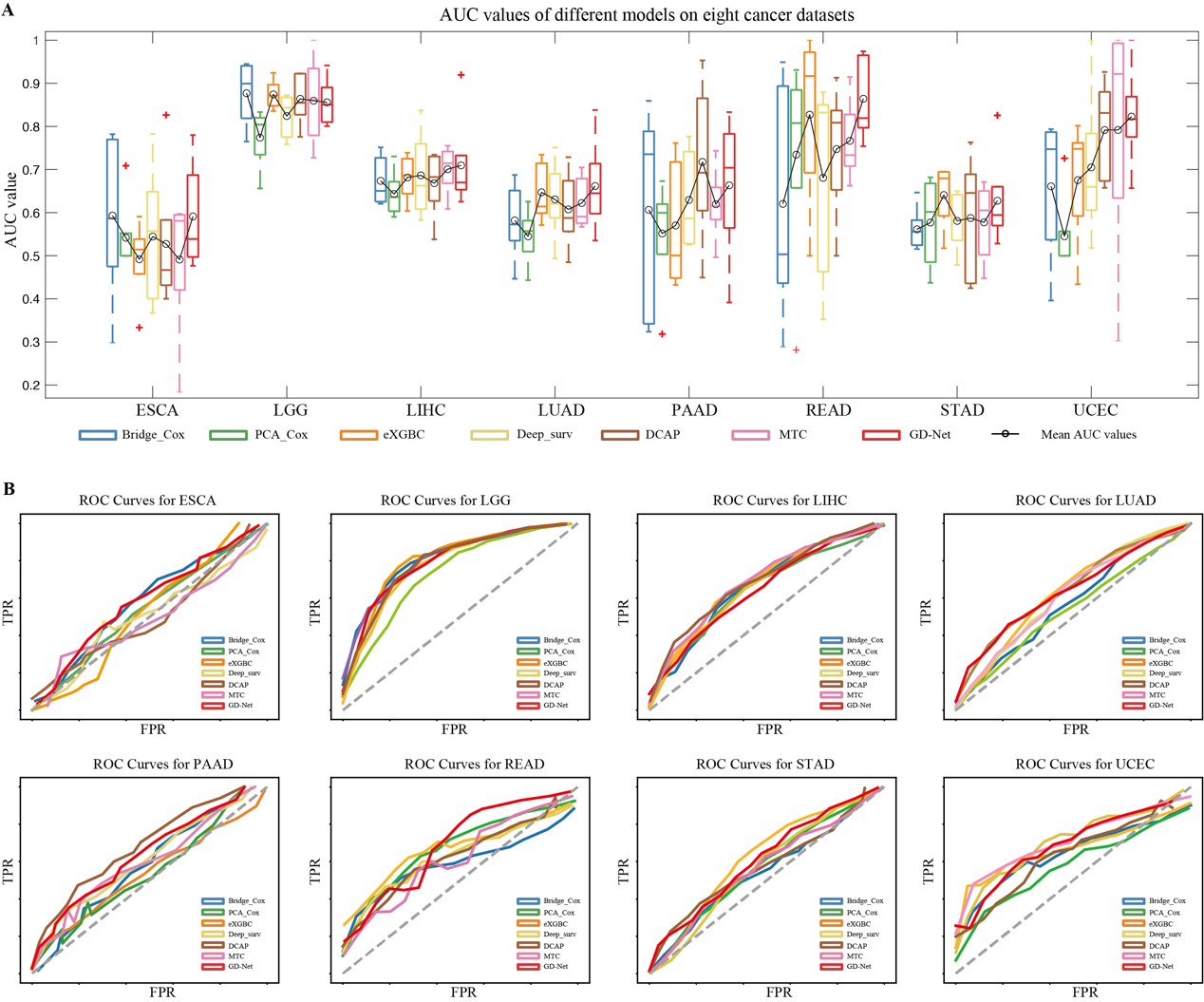


**Figure S1** Performance comparison with other models on eight cancer datasets based on AUC values.


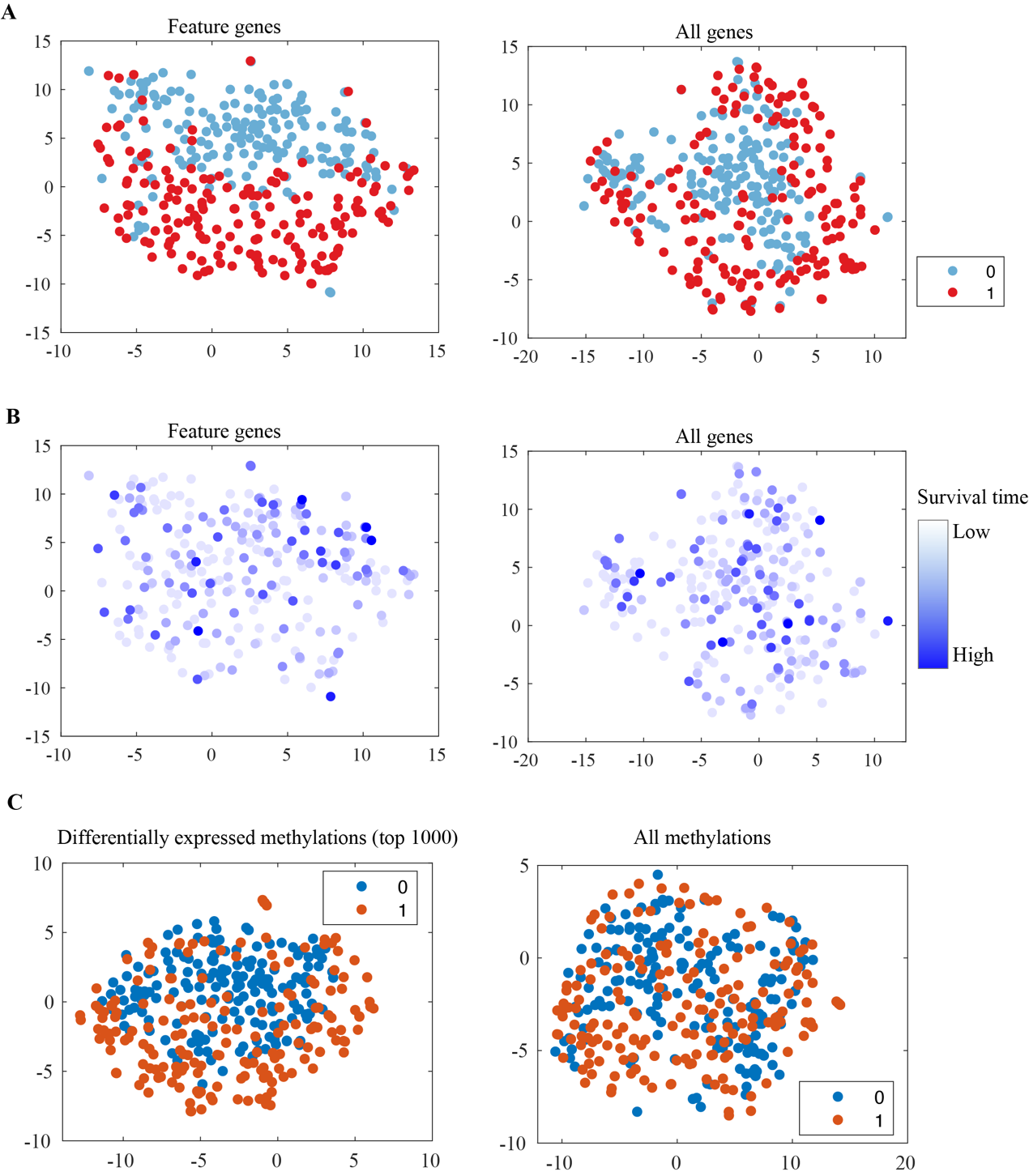


**Figure S2** GD-Net model accurately identifies informative genes. A The performance of TSNE clustering based on features selected by GD-Net and all genes. Each dot is a patient. B The result of TSNE clustering based on features selected by GD-Net and all genes and labeled by original survival time. C The clustering result of the differentially expressed methylations is displayed. All molecules are as shuffle group.


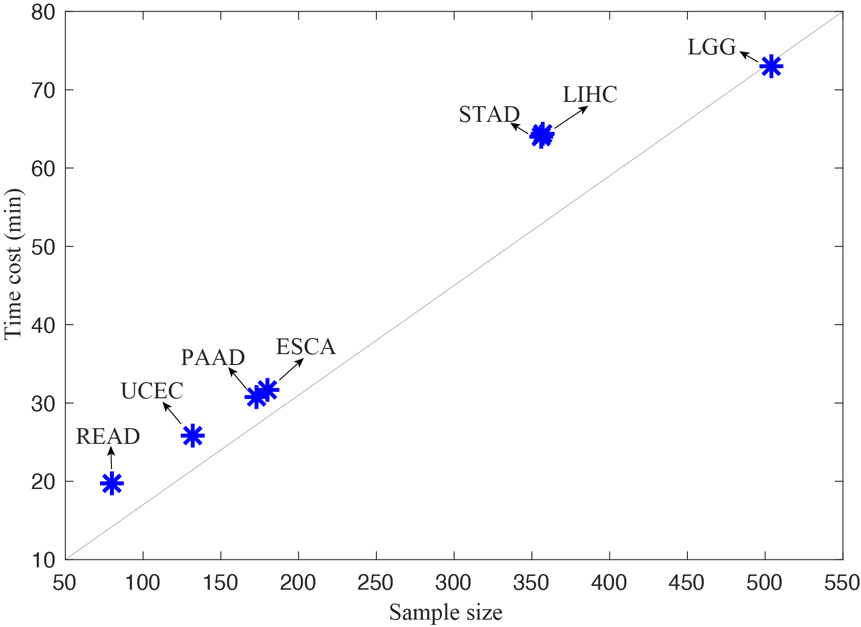


**Figure S3** Time cost of GD-Net on eight cancer datasets based on GPU platform.


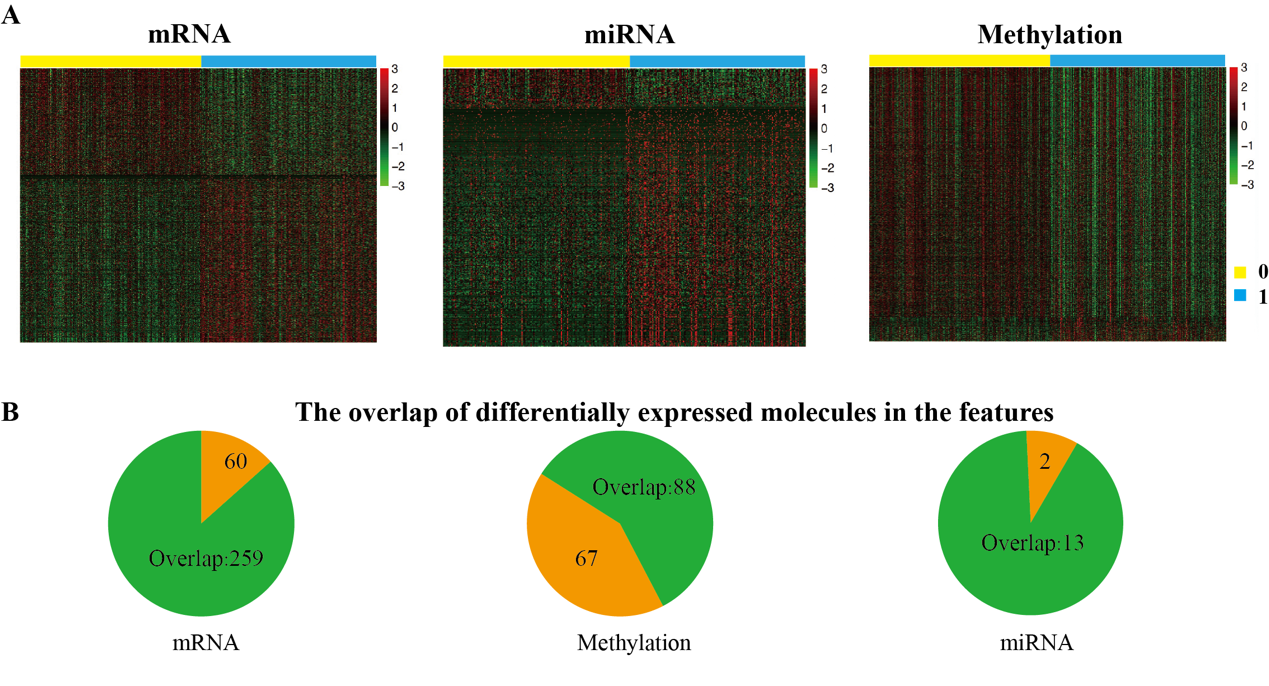


**Figure S4** Case study on liver cancer. A The hierarchical clustering of two predicted risk groups based on informative genes. B The overlap of differentially expressed molecules in the features.


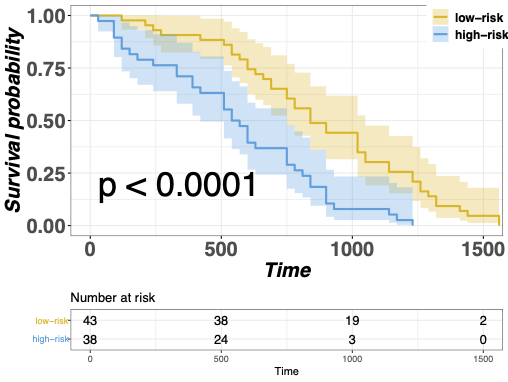


**Figure S5** The prediction results of additional datasets GSE54236. The Kaplan-Meier survival curves show the clinical relevance of the two predicted groups. The statistical p-values were determined by the two-tailed log-rank sum test.


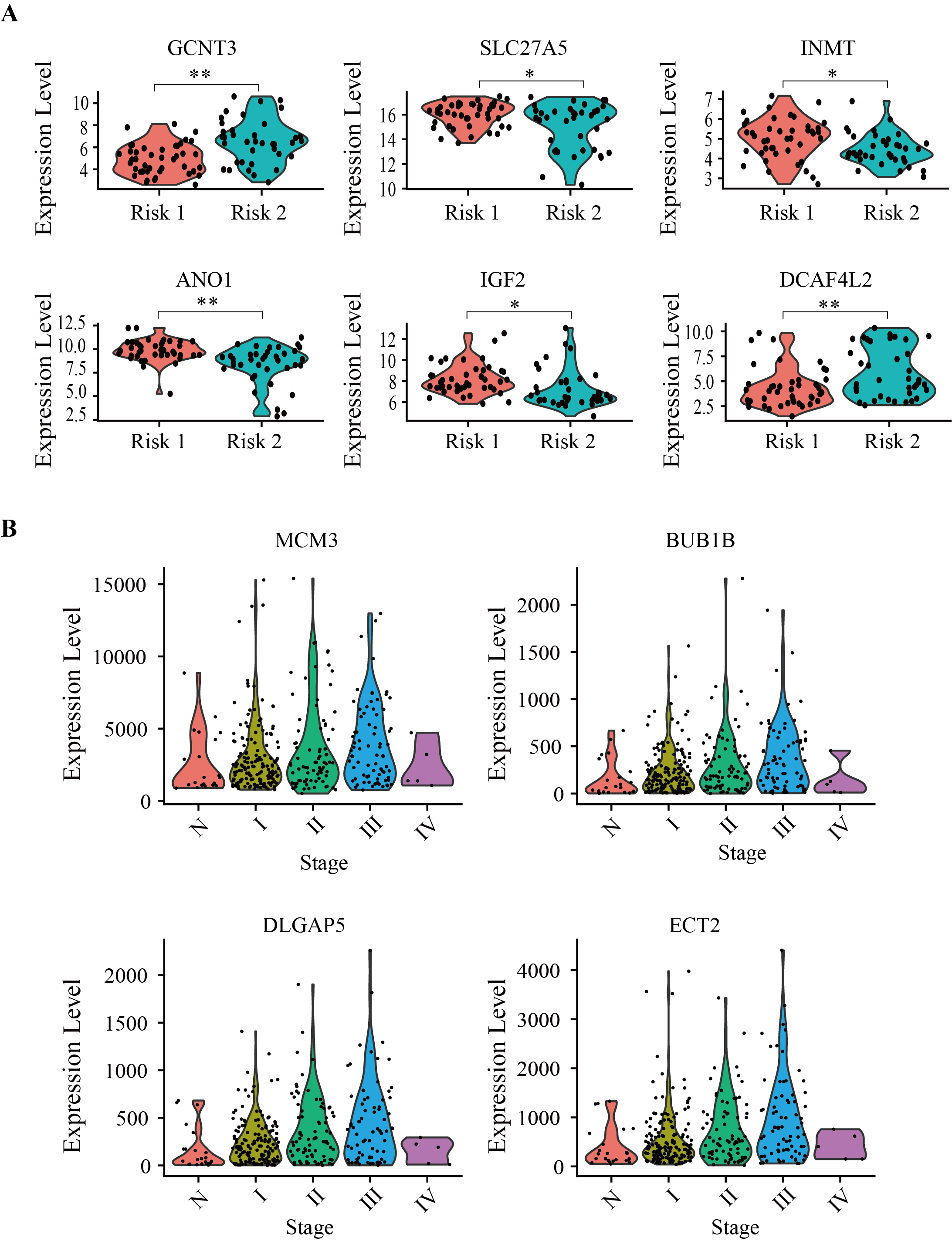


**Figure S6** Additional dataset validation of informative genes. A The expression level of informative genes between two risk groups. B Additional validation of informative genes.


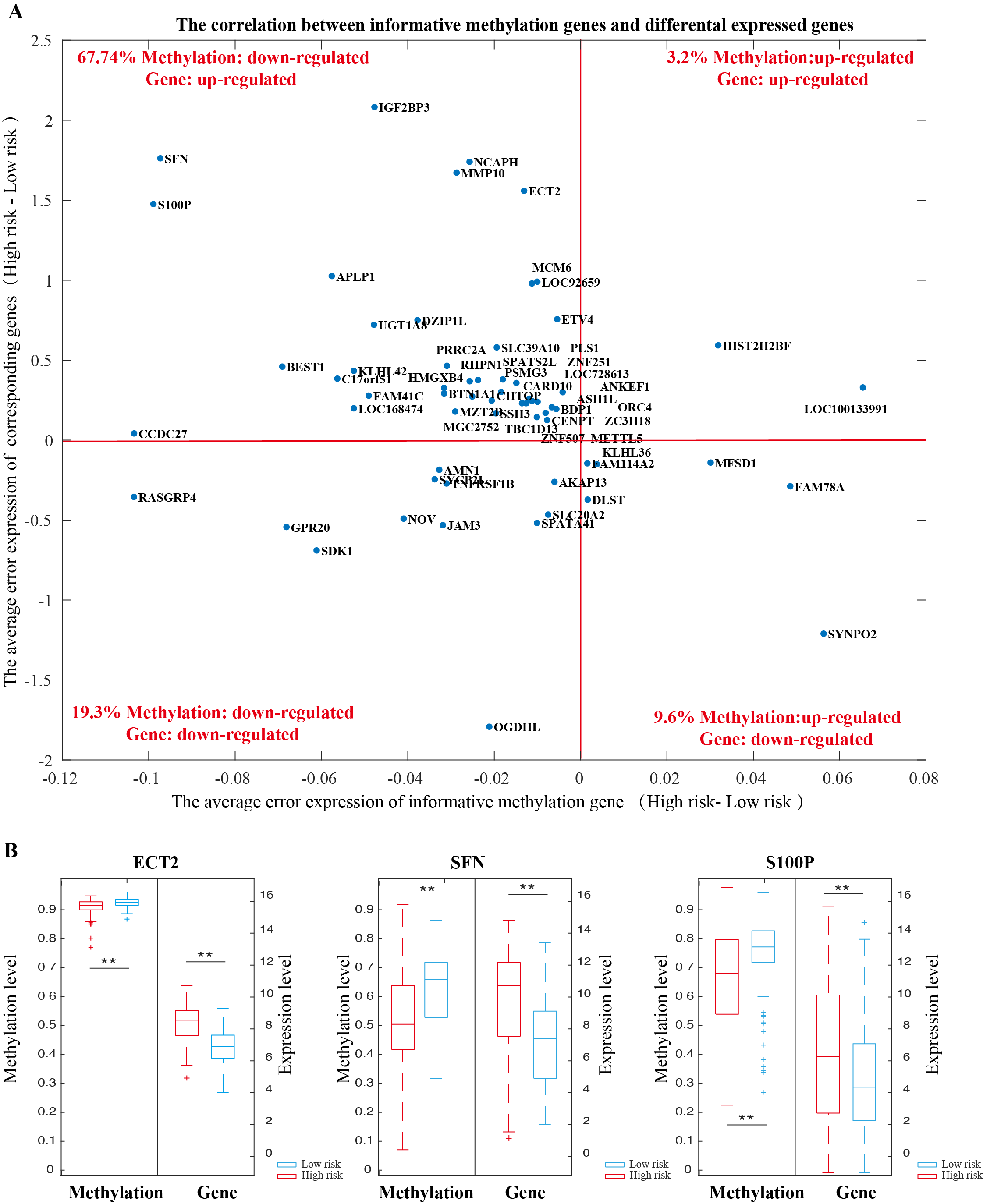


**Figure S7** Association analysis between informative methylated genes and relevant genes. A The expression correlation analysis between mRNA and methylation. B The expression level and methylation level of *ECT2, SFN* and *S100P* between two risk groups.


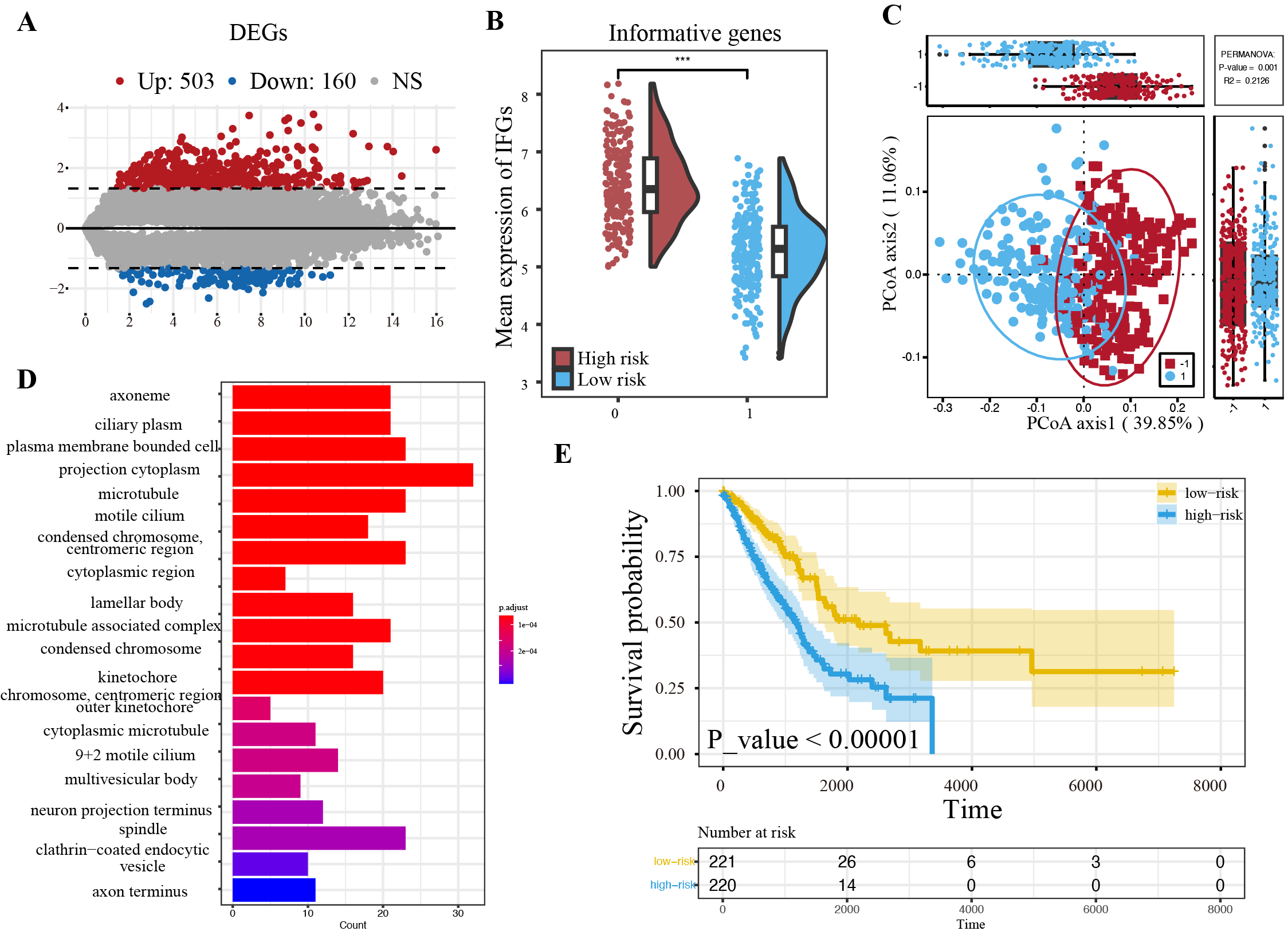


**Figure S8** Case study on Lung adenocarcinoma (LUAD). A. The differentially expressed genes for risk groups. The blue nodes represent down-regulated risk genes, and the red nodes indicate up-regulated risk ones (|log2FoldChange| >1.6 and corrected p-values <0.05). B. The half violin plot shows the average expression of informative genes between two risk groups. C. Quantification of the expression profile differences of informative genes between two risk groups by PCoA analysis. D. The enrichment bar plot shows the significantly enriched pathways of informative genes (FDR <0.05). E. The Kaplan-Meier survival curves show the clinical relevance of the two risk groups. The statistical p-values were determined by the two-tailed log-rank sum test.

**Table S1** The statistical information about the cancer experimental datasets

|  | Uncensored | | Censored | | Censored rate (%) | | Uncensored | | Censored | | Censored rate (%) | |  |
| --- | --- | --- | --- | --- | --- | --- | --- | --- | --- | --- | --- | --- | --- |
| ESCA | | 38 | | 231 | 85.87 | PAAD | | 92 | | 81 | | 46.82 | |
| LGG | | 124 | | 380 | 75.4 | READ | | 13 | | 67 | | 83.75 | |
| LIHC | | 123 | | 234 | 65.55 | STAD | | 140 | | 216 | | 60.67 | |
| LUAD | | 155 | | 286 | 64.85 | UCEC | | 23 | | 109 | | 82.57 | |
|  | |  | |  |  | Total | | 708 | | 1604 | | 69.38 | |
| *ESCA：Esophageal carcinoma; LGG: Brain Lower Grade Glioma; LIHC: Liver hepatocellular carcinoma; LUAD: Lung adenocarcinoma; PAAD: Pancreatic adenocarcinoma; READ: Rectum adenocarcinoma; STAD: Stomach adenocarcinoma; UCEC: Uterine Corpus Endometrial Carcinoma | | | | | | | | | | | | | |

**Table S2** The informative features selected by GD-Net

| XGB | Feature | Gain | Cover | Frequency |
| --- | --- | --- | --- | --- |
| 1 | EMCN_geneExp | 0.17193332 | 0.07834017 | 0.01581509 |
| 1 | CCL14_geneExp | 0.15611259 | 0.07223565 | 0.01581509 |
| 1 | CIP2A_geneExp | 0.03816022 | 0.02553327 | 0.00851582 |
| 1 | A1BG-AS1_geneExp | 0.02428212 | 0.02632295 | 0.01581509 |
| 1 | CKAP2L_geneExp | 0.01961392 | 0.01601491 | 0.00608273 |
| 1 | ORC6_geneExp | 0.01900332 | 0.01788269 | 0.00608273 |
| 1 | SPAG7_geneExp | 0.01694212 | 0.01235596 | 0.00608273 |
| 0 | SECISBP2L_methylation | 0.01514214 | 0.01726895 | 0.00729927 |
| 1 | SGO2_geneExp | 0.01451225 | 0.01944042 | 0.01094891 |
| 1 | ORC4_methylation | 0.01232762 | 0.01212927 | 0.00364964 |
| 1 | CATSPERZ_geneExp | 0.01126081 | 0.00907881 | 0.00243309 |
| 1 | SHCBP1_geneExp | 0.01094416 | 0.01236394 | 0.01216545 |
| 1 | B4GALT4_geneExp | 0.01074551 | 0.00184812 | 0.00243309 |
| 1 | MTMR9LP_geneExp | 0.01064966 | 0.00710615 | 0.00608273 |
| 0 | HIST1H4K_methylation | 0.0100001 | 0.01516475 | 0.00486618 |
| 1 | SRL_geneExp | 0.00939393 | 0.00994975 | 0.00364964 |
| 1 | G6PD_geneExp | 0.00883958 | 0.01462568 | 0.00851582 |
| 1 | DLGAP5_geneExp | 0.00843709 | 0.00273088 | 0.00364964 |
| 1 | OGDHL_methylation | 0.00836228 | 0.00111751 | 0.00121655 |
| 1 | PLD2_geneExp | 0.00776352 | 0.00301165 | 0.00364964 |
| 1 | CLEC3B_geneExp | 0.00745105 | 0.00813015 | 0.00243309 |
| 1 | SERPINF1_geneExp | 0.00715516 | 0.01252126 | 0.00729927 |
| 0 | KCTD15_geneExp | 0.00671919 | 0.00905113 | 0.00364964 |
| 1 | ZNF215_geneExp | 0.00657326 | 0.00161612 | 0.00121655 |
| 1 | CCM2L_geneExp | 0.00654345 | 0.01150191 | 0.01094891 |
| 1 | SLC39A10_methylation | 0.00572608 | 0.00838368 | 0.01094891 |
| 1 | RAMP3_geneExp | 0.00560949 | 0.00993433 | 0.00729927 |
| 1 | AMN1_methylation | 0.00552341 | 0.00140409 | 0.00243309 |
| 1 | LCN12_geneExp | 0.00544792 | 0.00861558 | 0.00608273 |
| 1 | NKAPD1_geneExp | 0.00490209 | 0.00207502 | 0.00243309 |
| 1 | RNF130_geneExp | 0.00488995 | 0.00428882 | 0.00121655 |
| 1 | ABCF1_geneExp | 0.00486906 | 0.00077646 | 0.00243309 |
| 1 | TRAIP_geneExp | 0.00478098 | 0.00468601 | 0.00121655 |
| 1 | PEX7_geneExp | 0.00446157 | 0.00093012 | 0.00121655 |
| 0 | RBAK_geneExp | 0.00445451 | 0.00066447 | 0.00121655 |
| 1 | INMT_geneExp | 0.00436938 | 0.00544146 | 0.00364964 |
| 1 | ZNF205-AS1_geneExp | 0.00435117 | 0.00434515 | 0.00121655 |
| 1 | CIT_geneExp | 0.00427908 | 0.00756415 | 0.00608273 |
| 1 | TTC9C_geneExp | 0.00418779 | 0.00691781 | 0.00973236 |
| 0 | CACNA2D4_geneExp | 0.00401016 | 0.00425413 | 0.00121655 |
| 1 | NUP88_geneExp | 0.00393605 | 0.00091919 | 0.00121655 |
| 1 | SNX15_geneExp | 0.0038822 | 0.00117126 | 0.00121655 |
| 0 | BCAM_methylation | 0.0038634 | 0.00521934 | 0.00486618 |
| 1 | ZC3H18_methylation | 0.00377978 | 0.00081507 | 0.00121655 |
| 1 | MIR3677_miRNAExp | 0.00371152 | 0.00520866 | 0.00243309 |
| 1 | BOLA3_geneExp | 0.00369684 | 0.00560632 | 0.00364964 |
| 1 | SHISA3_geneExp | 0.00359045 | 0.00396977 | 0.00121655 |
| 1 | HIST2H2BF_methylation | 0.00357091 | 0.00419067 | 0.00243309 |
| 1 | FGA_geneExp | 0.00356203 | 0.00399483 | 0.00121655 |
| 1 | TBC1D13_methylation | 0.00350154 | 0.00122792 | 0.00243309 |
| 1 | PGM5_geneExp | 0.00347974 | 0.00222598 | 0.00121655 |
| 0 | CLEC11A_methylation | 0.00342218 | 0.00359099 | 0.00121655 |
| 1 | CCDC69_geneExp | 0.0033105 | 0.00384861 | 0.00729927 |
| 1 | DDIAS_geneExp | 0.00321015 | 0.00410873 | 0.00121655 |
| 1 | SLC20A2_methylation | 0.00317094 | 0.00064231 | 0.00121655 |
| 1 | CHTOP_methylation | 0.00303973 | 0.00365134 | 0.00121655 |
| 1 | ECHDC2_geneExp | 0.00294698 | 0.00119468 | 0.00121655 |
| 1 | TMEM266_geneExp | 0.00293552 | 0.00397025 | 0.00121655 |
| 1 | HBD_geneExp | 0.00293469 | 0.0041076 | 0.00121655 |
| 1 | TWISTNB_geneExp | 0.00279924 | 0.00062863 | 0.00121655 |
| 0 | JAM3_methylation | 0.00277195 | 0.00345952 | 0.00121655 |
| 1 | SYNPO2_methylation | 0.00266391 | 0.00335085 | 0.00121655 |
| 1 | BLOC1S4_geneExp | 0.00260726 | 0.00373903 | 0.00364964 |
| 1 | HMGXB4_methylation | 0.00258663 | 0.00260258 | 0.00486618 |
| 1 | HMGA1_geneExp | 0.00251196 | 0.00090904 | 0.00121655 |
| 1 | CDK20_geneExp | 0.00237811 | 0.00145345 | 0.00243309 |
| 1 | FADS6_methylation | 0.00233236 | 0.00298892 | 0.00121655 |
| 0 | MRPS14_geneExp | 0.00230177 | 0.0010313 | 0.00121655 |
| 1 | GSE1_geneExp | 0.00226465 | 0.00092393 | 0.00121655 |
| 1 | MYOM1_geneExp | 0.00226089 | 0.00422187 | 0.00364964 |
| 1 | CDH23_geneExp | 0.00223281 | 0.00486895 | 0.00973236 |
| 1 | VPS28_methylation | 0.0021915 | 0.00382626 | 0.00486618 |
| 1 | CLEC9A_geneExp | 0.00216795 | 0.00386395 | 0.00121655 |
| 0 | MIR210_methylation | 0.00209489 | 0.00389618 | 0.00121655 |
| 1 | BUB1B_geneExp | 0.00208074 | 0.00327726 | 0.00121655 |
| 1 | GRAPL_geneExp | 0.00204393 | 0.00349577 | 0.00121655 |
| 1 | NADK2_geneExp | 0.00203628 | 0.00088202 | 0.00121655 |
| 1 | CSGALNACT2_methylation | 0.00203611 | 0.00312981 | 0.00121655 |
| 1 | SELE_geneExp | 0.00202967 | 0.00378062 | 0.00121655 |
| 1 | APMAP_geneExp | 0.00201904 | 0.00369801 | 0.00121655 |
| 1 | APOA5_geneExp | 0.00200102 | 0.00043098 | 0.00121655 |
| 1 | XIRP2_geneExp | 0.00197307 | 0.0039868 | 0.00121655 |
| 1 | S100P_methylation | 0.00195193 | 0.00130014 | 0.00243309 |
| 1 | PLS1_methylation | 0.00194655 | 0.00108513 | 0.00121655 |
| 1 | C7_geneExp | 0.00193958 | 0.00264982 | 0.00121655 |
| 1 | MIR29C_miRNAExp | 0.00193395 | 0.00286642 | 0.00121655 |
| 1 | ETFRF1_geneExp | 0.00188037 | 0.00095513 | 0.00121655 |
| 1 | PNMA5_geneExp | 0.0018574 | 0.00309114 | 0.00121655 |
| 1 | LOC728613_methylation | 0.00184795 | 0.00090705 | 0.00121655 |
| 0 | FDXACB1_geneExp | 0.00181911 | 0.00083567 | 0.00121655 |
| 1 | SHISAL1_geneExp | 0.0018123 | 0.00097867 | 0.00243309 |
| 1 | MFSD1_methylation | 0.00179377 | 0.00250508 | 0.00121655 |
| 1 | AIFM1_geneExp | 0.00175415 | 0.00048325 | 0.00121655 |
| 0 | FAM78A_methylation | 0.00174915 | 0.00234085 | 0.00121655 |
| 1 | SLC8B1_methylation | 0.00173477 | 0.00335528 | 0.00121655 |
| 1 | RPL36_methylation | 0.00173374 | 0.00218659 | 0.00121655 |
| 0 | ETV4_methylation | 0.00172084 | 0.00051096 | 0.00121655 |
| 0 | HIST2H2AC_methylation | 0.0016255 | 0.00134926 | 0.00121655 |
| 1 | MZT2B_methylation | 0.00153677 | 0.0007545 | 0.00121655 |
| 1 | CCDC27_methylation | 0.00153277 | 0.00255044 | 0.00243309 |
| 1 | CAPN3_geneExp | 0.00153173 | 0.00247707 | 0.00121655 |
| 1 | COL1A1_geneExp | 0.00151263 | 0.00328518 | 0.00121655 |
| 1 | DCN_geneExp | 0.00149964 | 0.00295021 | 0.00121655 |
| 1 | BPHL_geneExp | 0.00146516 | 0.00265797 | 0.00121655 |
| 0 | KHNYN_geneExp | 0.00145953 | 0.00266503 | 0.00121655 |
| 0 | PSAP_methylation | 0.00145007 | 0.00073728 | 0.00121655 |
| 0 | HIST1H4J_methylation | 0.00143421 | 0.00227692 | 0.00121655 |
| 1 | PURG_methylation | 0.00141692 | 0.0005497 | 0.00121655 |
| 1 | BTN1A1_methylation | 0.00140038 | 0.00107965 | 0.00121655 |
| 0 | CD101_geneExp | 0.00139713 | 0.00085929 | 0.00121655 |
| 1 | GLTPD2_geneExp | 0.00137346 | 0.00184642 | 0.00243309 |
| 0 | C1GALT1_methylation | 0.0013701 | 0.00353151 | 0.00121655 |
| 1 | KLHL42_methylation | 0.001331 | 0.00216108 | 0.00121655 |
| 1 | HEY1_geneExp | 0.00130483 | 0.0031508 | 0.00121655 |
| 1 | DCLK2_geneExp | 0.00130266 | 0.00174946 | 0.00121655 |
| 1 | ASGR2_geneExp | 0.0012922 | 0.00041903 | 0.00121655 |
| 1 | OTUD7B_methylation | 0.0012834 | 0.00274977 | 0.00608273 |
| 1 | LCORL_geneExp | 0.00125033 | 0.00075326 | 0.00121655 |
| 1 | CENPO_geneExp | 0.00123565 | 0.00067401 | 0.00121655 |
| 1 | HULC_geneExp | 0.00122679 | 0.00051025 | 0.00121655 |
| 1 | CD28_geneExp | 0.00122596 | 0.00204899 | 0.00364964 |
| 1 | HSPA12B_geneExp | 0.00122503 | 0.0005865 | 0.00121655 |
| 0 | C20orf202_geneExp | 0.00122448 | 0.00206325 | 0.00121655 |
| 1 | LHFPL4_geneExp | 0.00121365 | 0.00314636 | 0.00121655 |
| 1 | C1RL_geneExp | 0.00119521 | 0.00088506 | 0.00121655 |
| 0 | H2AFB1_geneExp | 0.00119177 | 0.00045738 | 0.00121655 |
| 1 | GIMAP1_geneExp | 0.00118811 | 0.00204656 | 0.00121655 |
| 1 | PSMG3_methylation | 0.00116025 | 0.00184889 | 0.00121655 |
| 1 | FATE1_geneExp | 0.00115187 | 0.00069218 | 0.00121655 |
| 1 | E2F6_geneExp | 0.00114267 | 0.00161677 | 0.00121655 |
| 1 | ZNF274_methylation | 0.00114009 | 0.00220677 | 0.00243309 |
| 1 | CARF_methylation | 0.00113244 | 0.00110152 | 0.00121655 |
| 1 | CLUH_geneExp | 0.00112527 | 0.00211501 | 0.00121655 |
| 1 | HTR1D_geneExp | 0.0011096 | 0.00176158 | 0.00243309 |
| 1 | CILP_geneExp | 0.00110879 | 0.00233967 | 0.00121655 |
| 1 | UBE2S_geneExp | 0.00109655 | 0.00208275 | 0.00121655 |
| 1 | WDR75_geneExp | 0.00108736 | 0.00264373 | 0.00486618 |
| 0 | RPRML_geneExp | 0.0010804 | 0.00139741 | 0.00121655 |
| 0 | VPS50_methylation | 0.00106589 | 0.00209076 | 0.00243309 |
| 0 | 45175_geneExp | 0.0010653 | 0.00110915 | 0.00121655 |
| 1 | ZNF90_geneExp | 0.00104554 | 0.00246003 | 0.00121655 |
| 0 | NBEAL1_methylation | 0.00103846 | 0.00181586 | 0.00243309 |
| 1 | LOC92659_methylation | 0.0010051 | 0.00081585 | 0.00121655 |
| 1 | SMIM7_geneExp | 0.00099637 | 0.00194207 | 0.00121655 |
| 1 | SGIP1_methylation | 0.00096872 | 0.00217677 | 0.00608273 |
| 1 | CR1_geneExp | 0.0009512 | 0.00061844 | 0.00121655 |
| 1 | BEST1_methylation | 0.00095018 | 0.00063399 | 0.00121655 |
| 1 | ASGR1_geneExp | 0.00094047 | 0.00461776 | 0.01216545 |
| 0 | AGAP1_methylation | 0.0009299 | 0.00038737 | 0.00121655 |
| 1 | AZU1_methylation | 0.00091881 | 0.00160491 | 0.00121655 |
| 0 | POU5F1_methylation | 0.00091635 | 0.00043713 | 0.00121655 |
| 1 | DZIP1L_methylation | 0.00091019 | 0.00208552 | 0.00121655 |
| 1 | MIR200C_miRNAExp | 0.0009053 | 0.00105114 | 0.00364964 |
| 1 | ECT2_geneExp | 0.00090034 | 0.00126 | 0.00121655 |
| 1 | APRT_methylation | 0.0008998 | 0.00174785 | 0.00121655 |
| 0 | SLC25A35_geneExp | 0.00089653 | 0.00069198 | 0.00121655 |
| 0 | ENAH_methylation | 0.00089611 | 0.00059115 | 0.00121655 |
| 1 | SZT2_geneExp | 0.00088994 | 0.00147076 | 0.00121655 |
| 0 | HSP90AB1_methylation | 0.00086151 | 0.00045627 | 0.00121655 |
| 1 | HGFAC_geneExp | 0.00086063 | 0.00283462 | 0.00364964 |
| 1 | A1BG_geneExp | 0.00085837 | 0.00046343 | 0.00121655 |
| 1 | NOVA2_geneExp | 0.00085184 | 0.00035102 | 0.00121655 |
| 1 | FAM41C_methylation | 0.00085178 | 0.0021029 | 0.00121655 |
| 1 | C17orf51_methylation | 0.00085088 | 0.00161157 | 0.00121655 |
| 1 | RAB7A_geneExp | 0.00084926 | 0.00043962 | 0.00121655 |
| 0 | TMEM74_geneExp | 0.00084693 | 0.00036839 | 0.00121655 |
| 0 | ACTR3B_geneExp | 0.00084047 | 0.00051272 | 0.00121655 |
| 0 | GJB7_methylation | 0.00083931 | 0.00247378 | 0.00121655 |
| 1 | NXPH4_geneExp | 0.00083876 | 0.00290897 | 0.00486618 |
| 1 | SPATS2L_methylation | 0.00083313 | 0.00252568 | 0.00121655 |
| 1 | C1orf174_geneExp | 0.00082885 | 0.00061217 | 0.00121655 |
| 0 | CLIP4_geneExp | 0.00081263 | 0.00052968 | 0.00121655 |
| 1 | ATF4_geneExp | 0.00080286 | 0.00152041 | 0.00486618 |
| 0 | LOC90586_methylation | 0.00078203 | 0.00120146 | 0.00121655 |
| 1 | RERE_geneExp | 0.0007799 | 0.00174548 | 0.00121655 |
| 0 | ABRAXAS1_geneExp | 0.00075084 | 0.00047182 | 0.00121655 |
| 1 | BLCAP_geneExp | 0.00073755 | 0.00329174 | 0.00121655 |
| 0 | SLFN12L_geneExp | 0.00073482 | 0.00051957 | 0.00121655 |
| 1 | CALCRL_geneExp | 0.00073481 | 0.00289953 | 0.00121655 |
| 1 | SSH3_methylation | 0.00071705 | 0.00045472 | 0.00121655 |
| 1 | SLC35F6_geneExp | 0.00071428 | 0.00138357 | 0.00121655 |
| 1 | CFHR1_geneExp | 0.00070058 | 0.00211854 | 0.00486618 |
| 0 | THAP12_geneExp | 0.00068888 | 0.00314235 | 0.00121655 |
| 0 | MMP10_methylation | 0.00068157 | 0.00105262 | 0.00121655 |
| 1 | RIT1_geneExp | 0.00067541 | 0.00138244 | 0.00243309 |
| 1 | PLCB1_geneExp | 0.00067514 | 0.00115673 | 0.00121655 |
| 1 | ASH1L_methylation | 0.0006727 | 0.00069941 | 0.00121655 |
| 1 | ETFDH_geneExp | 0.00065547 | 0.00057822 | 0.00121655 |
| 1 | ARHGEF15_geneExp | 0.00064594 | 0.00335627 | 0.00851582 |
| 1 | KMT5A_geneExp | 0.0006425 | 0.00053469 | 0.00121655 |
| 1 | HINT3_geneExp | 0.00063744 | 0.00168465 | 0.00486618 |
| 0 | ZFY_methylation | 0.0006342 | 0.0004885 | 0.00121655 |
| 1 | RABGGTA_geneExp | 0.0006241 | 0.00053093 | 0.00121655 |
| 1 | CPB2_geneExp | 0.00060463 | 0.00058053 | 0.00121655 |
| 1 | CXCL3_geneExp | 0.00060007 | 0.00314594 | 0.00121655 |
| 0 | MGC14436_geneExp | 0.00059951 | 0.00339697 | 0.00121655 |
| 1 | C10orf105_geneExp | 0.00059274 | 0.00218555 | 0.00364964 |
| 0 | TCAIM_methylation | 0.00058748 | 0.00047712 | 0.00121655 |
| 1 | SHMT1_geneExp | 0.00058204 | 0.0027131 | 0.00608273 |
| 0 | ARRDC1-AS1_methylation | 0.00058 | 0.00026186 | 0.00121655 |
| 1 | UGT1A8_methylation | 0.00057755 | 0.00056416 | 0.00121655 |
| 0 | TMEM45B_geneExp | 0.0005659 | 0.00110572 | 0.00121655 |
| 0 | RPS15_methylation | 0.000564 | 0.00035122 | 0.00121655 |
| 1 | PPP1R14B_geneExp | 0.0005531 | 0.0006071 | 0.00121655 |
| 0 | FAIM2_methylation | 0.00055157 | 0.00162496 | 0.00121655 |
| 1 | NCOA6_geneExp | 0.00054813 | 0.00070064 | 0.00121655 |
| 1 | CYP3A7_geneExp | 0.00054276 | 0.00122253 | 0.00121655 |
| 1 | RHPN1_methylation | 0.00054151 | 0.00158129 | 0.00121655 |
| 0 | PDZD9_geneExp | 0.00053872 | 0.00043735 | 0.00121655 |
| 1 | COPG1_geneExp | 0.00053862 | 0.00092256 | 0.00121655 |
| 0 | SGMS2_methylation | 0.00053439 | 0.00208674 | 0.00121655 |
| 1 | XCR1_geneExp | 0.00052875 | 0.00033727 | 0.00121655 |
| 1 | MRS2_geneExp | 0.00052572 | 0.00094902 | 0.00121655 |
| 0 | ADCK2_geneExp | 0.00051644 | 0.00036899 | 0.00121655 |
| 1 | MMRN2_geneExp | 0.00051341 | 0.00234707 | 0.00121655 |
| 1 | SLC52A2_geneExp | 0.00051335 | 0.00125314 | 0.00364964 |
| 0 | ANKRD1_methylation | 0.00051204 | 0.00125495 | 0.00121655 |
| 1 | TONSL_geneExp | 0.00051113 | 0.00136662 | 0.00243309 |
| 0 | SRM_methylation | 0.00050757 | 0.0005509 | 0.00121655 |
| 0 | ABCB7_geneExp | 0.0005021 | 0.00065111 | 0.00121655 |
| 1 | MIR519C_miRNAExp | 0.00050079 | 0.00168493 | 0.00486618 |
| 1 | OSMR_methylation | 0.0004901 | 0.0015192 | 0.00243309 |
| 1 | CD34_geneExp | 0.00049007 | 0.00122593 | 0.00243309 |
| 0 | CILP2_methylation | 0.00048382 | 0.00047296 | 0.00121655 |
| 0 | ZNF248_methylation | 0.00048024 | 0.00068717 | 0.00121655 |
| 0 | PLAA_methylation | 0.00047623 | 0.00251608 | 0.00121655 |
| 1 | CREB3L4_geneExp | 0.00046767 | 0.00056355 | 0.00121655 |
| 1 | HES2_geneExp | 0.00045639 | 0.00114724 | 0.00243309 |
| 0 | ADGRE5_geneExp | 0.00045343 | 0.00033094 | 0.00121655 |
| 1 | MIR450A-1_miRNAExp | 0.00044473 | 0.0004642 | 0.00121655 |
| 0 | GLP1R_methylation | 0.00044466 | 0.00066049 | 0.00121655 |
| 1 | LOC654342_geneExp | 0.00044275 | 0.00133043 | 0.00243309 |
| 0 | MIR4734_miRNAExp | 0.00043982 | 0.00130394 | 0.00121655 |
| 1 | AAMDC_geneExp | 0.00043948 | 0.00053283 | 0.00121655 |
| 1 | SS18L2_methylation | 0.00043845 | 0.00086251 | 0.00121655 |
| 1 | DDX55_geneExp | 0.00043288 | 0.0008315 | 0.00121655 |
| 1 | NCDN_geneExp | 0.00043244 | 0.00074898 | 0.00121655 |
| 1 | MCM3_geneExp | 0.00043196 | 0.00048214 | 0.00121655 |
| 0 | TMEM25_methylation | 0.00041748 | 0.00088815 | 0.00121655 |
| 0 | CDC7_methylation | 0.00041734 | 0.0003608 | 0.00121655 |
| 0 | PIP4P1_geneExp | 0.00041298 | 0.0017345 | 0.00121655 |
| 1 | TCL6_geneExp | 0.00041281 | 0.0007274 | 0.00121655 |
| 0 | IBA57_methylation | 0.00040911 | 0.0012873 | 0.00121655 |
| 1 | SNORA77_methylation | 0.00040763 | 0.00030725 | 0.00121655 |
| 1 | KLRK1_geneExp | 0.00039755 | 0.00040169 | 0.00121655 |
| 1 | TACC2_methylation | 0.00039634 | 0.00221818 | 0.00608273 |
| 1 | LMOD1_geneExp | 0.00039476 | 0.00114941 | 0.00486618 |
| 1 | NFKBIA_geneExp | 0.00039185 | 0.00057195 | 0.00121655 |
| 1 | ADCYAP1_geneExp | 0.00039013 | 0.00263377 | 0.00729927 |
| 0 | DHRS7_methylation | 0.0003892 | 0.00029871 | 0.00121655 |
| 1 | RHOC_methylation | 0.00038899 | 0.00122037 | 0.00243309 |
| 0 | BIK_geneExp | 0.0003884 | 0.00064629 | 0.00121655 |
| 0 | ANKRD16_methylation | 0.00038538 | 0.00058319 | 0.00121655 |
| 1 | APOC3_geneExp | 0.00038493 | 0.00088018 | 0.00121655 |
| 1 | SSR3_geneExp | 0.0003777 | 0.00036544 | 0.00121655 |
| 1 | IKZF3_methylation | 0.00037165 | 0.00049246 | 0.00121655 |
| 1 | EHD2_geneExp | 0.00036169 | 0.00193993 | 0.00121655 |
| 1 | LOC728989_geneExp | 0.00036082 | 0.00069214 | 0.00243309 |
| 1 | ABCC6_geneExp | 0.0003524 | 0.00148361 | 0.00121655 |
| 1 | CTSH_geneExp | 0.00035112 | 0.00075219 | 0.00121655 |
| 0 | AARSD1_geneExp | 0.00035046 | 0.00027224 | 0.00121655 |
| 1 | CHAF1B_geneExp | 0.00034655 | 0.00027633 | 0.00121655 |
| 1 | ESR1_geneExp | 0.00034532 | 0.00109498 | 0.00121655 |
| 1 | FOLR2_geneExp | 0.00034265 | 0.00036077 | 0.00121655 |
| 1 | KLHL28_methylation | 0.00034147 | 0.00144214 | 0.00243309 |
| 1 | RAMP2_geneExp | 0.0003414 | 0.00079895 | 0.00121655 |
| 0 | MAPK1IP1L_geneExp | 0.00034107 | 0.001338 | 0.00121655 |
| 1 | CYSTM1_geneExp | 0.00033452 | 0.0006162 | 0.00121655 |
| 0 | TRADD_geneExp | 0.00033274 | 0.00067557 | 0.00121655 |
| 1 | AUNIP_geneExp | 0.00032925 | 0.00096273 | 0.00243309 |
| 0 | C1QTNF6_geneExp | 0.00032604 | 0.00033798 | 0.00121655 |
| 1 | INCA1_geneExp | 0.00032516 | 0.00179413 | 0.00486618 |
| 1 | CARD10_methylation | 0.00032365 | 0.00161345 | 0.00364964 |
| 0 | LYPD6B_methylation | 0.00032248 | 0.00110164 | 0.00121655 |
| 1 | ADCY4_geneExp | 0.00031597 | 0.00123512 | 0.00364964 |
| 0 | FAM193B_geneExp | 0.00031545 | 0.00040953 | 0.00121655 |
| 1 | SPP2_geneExp | 0.00031312 | 0.00127832 | 0.00243309 |
| 1 | MIR891A_miRNAExp | 0.00031268 | 0.00178125 | 0.00608273 |
| 1 | SMAD1_geneExp | 0.00031254 | 0.00069614 | 0.00121655 |
| 1 | ZNF630_geneExp | 0.00031231 | 0.00031423 | 0.00121655 |
| 0 | TAB1_geneExp | 0.00030975 | 0.00169671 | 0.00121655 |
| 1 | ZNF595_geneExp | 0.00030735 | 0.00096065 | 0.00121655 |
| 1 | MIR520H_miRNAExp | 0.00030688 | 0.0006277 | 0.00121655 |
| 1 | MIR526B_miRNAExp | 0.00030644 | 0.00086284 | 0.00121655 |
| 1 | FAM118B_geneExp | 0.00030484 | 0.0006358 | 0.00121655 |
| 0 | KIF1A_geneExp | 0.00030436 | 0.00039436 | 0.00121655 |
| 0 | PRSS21_geneExp | 0.00030231 | 0.00034022 | 0.00121655 |
| 0 | JADE3_methylation | 0.00030019 | 0.00096326 | 0.00364964 |
| 1 | NECTIN1_geneExp | 0.00029824 | 0.00059692 | 0.00121655 |
| 0 | NECAP1_methylation | 0.00029815 | 0.00054493 | 0.00121655 |
| 0 | LGALS8_geneExp | 0.00029476 | 0.00024458 | 0.00121655 |
| 1 | SEMA4G_methylation | 0.00029354 | 0.000481 | 0.00121655 |
| 1 | CASP12_geneExp | 0.00029137 | 0.0003347 | 0.00121655 |
| 1 | ECT2_methylation | 0.00028507 | 0.00087581 | 0.00243309 |
| 1 | IGF2_geneExp | 0.00028165 | 0.00085024 | 0.00121655 |
| 1 | SLC1A4_geneExp | 0.00027923 | 0.00047142 | 0.00121655 |
| 1 | DDX10_geneExp | 0.00027584 | 0.00138467 | 0.00486618 |
| 1 | ANXA13_geneExp | 0.00027302 | 0.00144029 | 0.00121655 |
| 1 | ASPDH_geneExp | 0.00026882 | 0.00103447 | 0.00121655 |
| 1 | CMTM2_methylation | 0.00026809 | 0.00135814 | 0.00486618 |
| 0 | AP1M1_geneExp | 0.00026625 | 0.00038743 | 0.00121655 |
| 1 | LTK_geneExp | 0.00026601 | 0.00077159 | 0.00121655 |
| 1 | PDSS2_geneExp | 0.00026556 | 0.00063379 | 0.00121655 |
| 1 | HIST2H3C_geneExp | 0.00026495 | 0.00098683 | 0.00364964 |
| 1 | ARPIN_geneExp | 0.0002621 | 0.00024989 | 0.00121655 |
| 1 | SLC7A11_geneExp | 0.00026074 | 0.00141113 | 0.00364964 |
| 1 | ZMYND8_geneExp | 0.00025583 | 0.00050925 | 0.00121655 |
| 1 | TMEM38B_geneExp | 0.0002557 | 0.00051033 | 0.00121655 |
| 1 | ADORA2A-AS1_geneExp | 0.00025312 | 0.00039027 | 0.00121655 |
| 1 | D2HGDH_geneExp | 0.00024745 | 0.00070502 | 0.00121655 |
| 1 | MIR3127_miRNAExp | 0.00024083 | 0.0006398 | 0.00121655 |
| 1 | CLSPN_geneExp | 0.00023891 | 0.00078168 | 0.00121655 |
| 1 | ANKFY1_geneExp | 0.00023702 | 0.00071827 | 0.00121655 |
| 1 | TNFRSF1B_methylation | 0.00023696 | 0.00058384 | 0.00121655 |
| 1 | KLK9_methylation | 0.0002319 | 0.00070481 | 0.00121655 |
| 1 | GPR20_methylation | 0.00022127 | 0.00062412 | 0.00121655 |
| 1 | LOC728758_methylation | 0.0002212 | 0.00080691 | 0.00121655 |
| 1 | FTH1_methylation | 0.00021782 | 0.00083307 | 0.00121655 |
| 1 | PTPN23_geneExp | 0.00021618 | 0.00054886 | 0.00121655 |
| 1 | GJD3_geneExp | 0.00021498 | 0.00053534 | 0.00121655 |
| 1 | MASTL_geneExp | 0.00021353 | 0.00056827 | 0.00121655 |
| 0 | TNN_geneExp | 0.00021246 | 0.00036193 | 0.00121655 |
| 1 | TARS2_geneExp | 0.00021183 | 0.00029259 | 0.00121655 |
| 0 | AFG3L1P_geneExp | 0.0002118 | 0.00027719 | 0.00121655 |
| 1 | TMEM97_geneExp | 0.00021095 | 0.0011381 | 0.00121655 |
| 1 | NOV_methylation | 0.00020617 | 0.00029881 | 0.00121655 |
| 1 | DDX39A_geneExp | 0.00020292 | 0.00048157 | 0.00121655 |
| 0 | NOXO1_geneExp | 0.00020076 | 0.00040505 | 0.00121655 |
| 1 | BDP1_methylation | 0.00019974 | 0.0002801 | 0.00121655 |
| 0 | MAPKAPK3_methylation | 0.00019762 | 0.00067214 | 0.00243309 |
| 1 | PDLIM2_geneExp | 0.00019652 | 0.00055716 | 0.00121655 |
| 0 | ZNF132_geneExp | 0.00019555 | 0.00029448 | 0.00121655 |
| 1 | SYCP2L_methylation | 0.00019389 | 0.00137237 | 0.00486618 |
| 1 | BTD_geneExp | 0.00019376 | 0.00060351 | 0.00121655 |
| 1 | TMEM175_geneExp | 0.00019371 | 0.00053953 | 0.00121655 |
| 1 | SPINK4_geneExp | 0.00019325 | 0.00078353 | 0.00121655 |
| 1 | PALM2-AKAP2_geneExp | 0.00019057 | 0.00046878 | 0.00121655 |
| 0 | LOC168474_methylation | 0.00018871 | 0.00045986 | 0.00121655 |
| 0 | LOC391322_geneExp | 0.00018659 | 0.00040584 | 0.00121655 |
| 1 | FGD5_geneExp | 0.00018554 | 0.00041714 | 0.00121655 |
| 1 | HAPLN1_geneExp | 0.00018416 | 0.00116971 | 0.00364964 |
| 1 | AKAP13_methylation | 0.00018385 | 0.00038556 | 0.00121655 |
| 1 | CCDC28A_geneExp | 0.00018218 | 0.0011665 | 0.00364964 |
| 1 | PEAR1_geneExp | 0.00017792 | 0.00128375 | 0.00121655 |
| 1 | RDH8_geneExp | 0.00017722 | 0.00231873 | 0.00121655 |
| 1 | TEX10_geneExp | 0.00016251 | 0.001424 | 0.00608273 |
| 0 | ABCA3_geneExp | 0.0001616 | 0.0003304 | 0.00121655 |
| 1 | MED18_methylation | 0.00015891 | 0.00068045 | 0.00243309 |
| 1 | RASGRP4_methylation | 0.00015803 | 0.00041385 | 0.00121655 |
| 0 | CKAP2L_methylation | 0.00015632 | 0.00048853 | 0.00121655 |
| 0 | ZNF785_methylation | 0.00015576 | 0.00042728 | 0.00121655 |
| 1 | GJA4_geneExp | 0.00015567 | 0.00045147 | 0.00121655 |
| 1 | GOLGA8A_methylation | 0.00015467 | 0.00037154 | 0.00121655 |
| 0 | VXN_methylation | 0.00015454 | 0.00034412 | 0.00121655 |
| 1 | ZNF426_geneExp | 0.00015399 | 0.0003234 | 0.00121655 |
| 1 | IGF2BP3_methylation | 0.00015199 | 0.00087109 | 0.00243309 |
| 1 | OGDHL_geneExp | 0.00015179 | 0.00062293 | 0.00121655 |
| 1 | PLEKHA3_methylation | 0.0001513 | 0.00034283 | 0.00121655 |
| 1 | GSTM5_geneExp | 0.00014943 | 0.00105677 | 0.00364964 |
| 0 | MIR6757_miRNAExp | 0.00014942 | 0.00040166 | 0.00121655 |
| 1 | RHEB_geneExp | 0.00014668 | 0.00030075 | 0.00121655 |
| 0 | UBE2A_geneExp | 0.00014463 | 0.00029158 | 0.00121655 |
| 1 | TRIP13_geneExp | 0.00014295 | 0.00039626 | 0.00121655 |
| 1 | PUS7_geneExp | 0.00013899 | 0.00031332 | 0.00121655 |
| 1 | CYP2U1_geneExp | 0.00013385 | 0.00039867 | 0.00121655 |
| 1 | SMYD5_geneExp | 0.0001335 | 0.00058905 | 0.00243309 |
| 0 | ZMAT2_geneExp | 0.00013336 | 0.00036562 | 0.00121655 |
| 1 | ZNF507_methylation | 0.00013289 | 0.00031192 | 0.00121655 |
| 1 | DTD1_geneExp | 0.00013119 | 0.00036987 | 0.00121655 |
| 1 | SOCS7_geneExp | 0.00013073 | 0.00080834 | 0.00243309 |
| 1 | MIR4510_miRNAExp | 0.00012843 | 0.00061151 | 0.00243309 |
| 1 | SLITRK5_methylation | 0.0001281 | 0.001132 | 0.00486618 |
| 1 | ZNF251_methylation | 0.00012622 | 0.00082736 | 0.00364964 |
| 1 | ANO7_geneExp | 0.00012553 | 0.00033944 | 0.00121655 |
| 0 | GPATCH1_methylation | 0.00012473 | 0.00111905 | 0.00486618 |
| 0 | IGFBP4_methylation | 0.00012427 | 0.00043729 | 0.00121655 |
| 0 | CNPY3_methylation | 0.00012236 | 0.00036113 | 0.00121655 |
| 1 | SFN_methylation | 0.0001193 | 0.00053776 | 0.00121655 |
| 0 | XPNPEP3_geneExp | 0.00011754 | 0.0003864 | 0.00121655 |
| 0 | KRT75_geneExp | 0.00011178 | 0.00258018 | 0.00121655 |
| 0 | ACOT9_methylation | 0.00011175 | 0.00034089 | 0.00121655 |
| 0 | WIPF1_methylation | 0.00011067 | 0.00034268 | 0.00121655 |
| 1 | LMO2_geneExp | 0.00011002 | 0.00056778 | 0.00243309 |
| 1 | TAGLN_geneExp | 0.00010913 | 0.00047578 | 0.00121655 |
| 0 | FAM114A2_methylation | 0.00010806 | 0.00105393 | 0.00486618 |
| 0 | MTSS1L_geneExp | 0.00010759 | 0.00034358 | 0.00121655 |
| 0 | EMILIN3_geneExp | 0.00010677 | 0.00087769 | 0.00121655 |
| 1 | EXOC3L2_geneExp | 0.00010571 | 0.00062428 | 0.00121655 |
| 1 | FLVCR1_geneExp | 0.0001055 | 0.00044121 | 0.00121655 |
| 1 | PPP1R12B_geneExp | 0.00010458 | 0.00030882 | 0.00121655 |
| 1 | AIF1L_geneExp | 0.00010418 | 0.0007168 | 0.00243309 |
| 1 | KCTD17_geneExp | 0.00010282 | 0.0002917 | 0.00121655 |
| 0 | HIST1H2BC_geneExp | 9.89E-05 | 0.00032734 | 0.00121655 |
| 1 | GAS6_geneExp | 9.75E-05 | 0.00041641 | 0.00121655 |
| 0 | MON2_methylation | 9.60E-05 | 0.00043814 | 0.00121655 |
| 1 | MIR518B_miRNAExp | 9.56E-05 | 0.00033066 | 0.00121655 |
| 1 | DLST_methylation | 9.48E-05 | 0.00037212 | 0.00121655 |
| 0 | TAF1B_methylation | 9.34E-05 | 0.0010067 | 0.00121655 |
| 1 | AFM_geneExp | 9.18E-05 | 0.00037808 | 0.00121655 |
| 1 | BRI3BP_geneExp | 9.08E-05 | 0.00066555 | 0.00243309 |
| 1 | STC2_geneExp | 9.00E-05 | 0.0003382 | 0.00121655 |
| 1 | ANXA7_geneExp | 8.91E-05 | 0.0008238 | 0.00364964 |
| 1 | SERPINA11_geneExp | 8.77E-05 | 0.0003409 | 0.00121655 |
| 1 | DNASE1L3_geneExp | 8.49E-05 | 0.00029803 | 0.00121655 |
| 1 | GARS_geneExp | 8.40E-05 | 0.00062093 | 0.00243309 |
| 1 | OSR2_geneExp | 8.23E-05 | 0.00079534 | 0.00364964 |
| 1 | ZNF611_geneExp | 8.23E-05 | 0.00034172 | 0.00121655 |
| 1 | ADM_geneExp | 8.10E-05 | 0.00037036 | 0.00121655 |
| 0 | PPP1R2_methylation | 7.90E-05 | 0.00025915 | 0.00121655 |
| 1 | ZC3H3_geneExp | 7.88E-05 | 0.00029494 | 0.00121655 |
| 0 | ANKEF1_methylation | 7.80E-05 | 0.00027326 | 0.00121655 |
| 1 | DENR_geneExp | 7.79E-05 | 0.00024509 | 0.00121655 |
| 1 | DLC1_geneExp | 7.74E-05 | 0.00029936 | 0.00121655 |
| 1 | SLCO2A1_geneExp | 7.62E-05 | 0.00028677 | 0.00121655 |
| 1 | MAP3K5_geneExp | 7.59E-05 | 0.00029571 | 0.00121655 |
| 1 | PRRC2A_methylation | 7.32E-05 | 0.00029546 | 0.00121655 |
| 0 | PLA2G16_methylation | 7.25E-05 | 0.00026549 | 0.00121655 |
| 0 | MAT1A_methylation | 7.18E-05 | 0.00025218 | 0.00121655 |
| 1 | SPATA41_methylation | 6.97E-05 | 0.00028149 | 0.00121655 |
| 0 | TTC24_geneExp | 6.92E-05 | 0.00028883 | 0.00121655 |
| 0 | SDHAF1_methylation | 6.90E-05 | 0.00025756 | 0.00121655 |
| 1 | MGC2752_methylation | 6.84E-05 | 0.00027416 | 0.00121655 |
| 1 | FTSJ3_geneExp | 6.71E-05 | 0.00099083 | 0.00121655 |
| 1 | FCGRT_geneExp | 6.65E-05 | 0.00051958 | 0.00243309 |
| 1 | DUSP5P1_geneExp | 6.57E-05 | 0.00029579 | 0.00121655 |
| 0 | FRMD3_methylation | 6.55E-05 | 0.00027883 | 0.00121655 |
| 0 | UBXN1_methylation | 6.40E-05 | 0.00056545 | 0.00243309 |
| 1 | CBLN3_geneExp | 6.37E-05 | 0.0004165 | 0.00121655 |
| 1 | RCC1_geneExp | 6.34E-05 | 0.00025732 | 0.00121655 |
| 1 | LOC100133991_methylation | 6.28E-05 | 0.00039606 | 0.00121655 |
| 1 | NCAPH_methylation | 6.19E-05 | 0.00026129 | 0.00121655 |
| 0 | SYT12_geneExp | 6.18E-05 | 0.00039266 | 0.00121655 |
| 1 | OIP5_geneExp | 6.16E-05 | 0.00027653 | 0.00121655 |
| 1 | CSTF3_geneExp | 6.00E-05 | 0.00027995 | 0.00121655 |
| 1 | 44986_geneExp | 5.96E-05 | 0.00113066 | 0.00121655 |
| 1 | TMPRSS9_geneExp | 5.73E-05 | 0.00054045 | 0.00243309 |
| 1 | APLP1_methylation | 5.71E-05 | 0.00025066 | 0.00121655 |
| 1 | DDAH1_methylation | 5.55E-05 | 0.00145939 | 0.00121655 |
| 1 | CDPF1_methylation | 5.54E-05 | 0.000533 | 0.00243309 |
| 0 | ZNF788P_geneExp | 5.43E-05 | 0.00024223 | 0.00121655 |
| 0 | MMP24_methylation | 5.25E-05 | 0.00051765 | 0.00243309 |
| 0 | TMEM92_geneExp | 5.25E-05 | 0.00051645 | 0.00243309 |
| 1 | FAM99A_geneExp | 5.21E-05 | 0.00035755 | 0.00121655 |
| 1 | SLC9A3R1_geneExp | 5.01E-05 | 0.00025047 | 0.00121655 |
| 1 | TPRXL_geneExp | 5.00E-05 | 0.00035311 | 0.00121655 |
| 1 | ARHGEF3_geneExp | 4.85E-05 | 0.00024393 | 0.00121655 |
| 0 | SNORD55_methylation | 4.79E-05 | 0.00034463 | 0.00121655 |
| 1 | S100P_geneExp | 4.66E-05 | 0.00039492 | 0.00121655 |
| 1 | UBE2MP1_geneExp | 4.50E-05 | 0.00025035 | 0.00121655 |
| 1 | SDK1_methylation | 4.43E-05 | 0.00033255 | 0.00121655 |
| 1 | METTL5_methylation | 4.25E-05 | 0.00032749 | 0.00121655 |
| 1 | PAK4_geneExp | 4.19E-05 | 0.00047044 | 0.00121655 |
| 0 | ABHD13_geneExp | 4.15E-05 | 0.00363201 | 0.00121655 |
| 1 | CYP27A1_geneExp | 4.02E-05 | 0.00031663 | 0.00121655 |
| 0 | BET1L_geneExp | 3.93E-05 | 0.00037391 | 0.00121655 |
| 0 | ANKRD42_methylation | 3.92E-05 | 0.00031312 | 0.00121655 |
| 1 | CENPT_methylation | 3.90E-05 | 0.00024322 | 0.00121655 |
| 1 | DHRS13_geneExp | 3.72E-05 | 0.00030533 | 0.00121655 |
| 0 | MIR371A_methylation | 3.68E-05 | 0.00045753 | 0.00121655 |
| 1 | WBP1L_geneExp | 3.68E-05 | 0.00030424 | 0.00121655 |
| 1 | DNALI1_methylation | 3.59E-05 | 0.00030108 | 0.00121655 |
| 1 | DNMBP_geneExp | 3.58E-05 | 0.0004183 | 0.00121655 |
| 1 | KLHL36_methylation | 3.54E-05 | 0.00029694 | 0.00121655 |
| 1 | MCM6_methylation | 3.51E-05 | 0.00029592 | 0.00121655 |
| 0 | FAM20B_geneExp | 3.40E-05 | 0.00046742 | 0.00121655 |
| 0 | SMYD2_methylation | 3.39E-05 | 0.00027462 | 0.00121655 |
| 1 | CPEB2_methylation | 3.28E-05 | 0.00045035 | 0.00121655 |
| 1 | KCNJ16_geneExp | 3.14E-05 | 0.00028131 | 0.00121655 |
| 1 | MIR5698_miRNAExp | 3.08E-05 | 0.00028041 | 0.00121655 |
| 1 | MIR147B_miRNAExp | 3.06E-05 | 0.00041815 | 0.00121655 |
| 1 | DNER_geneExp | 3.02E-05 | 0.0002768 | 0.00121655 |
| 1 | GRK5_geneExp | 2.85E-05 | 0.00026819 | 0.00121655 |
| 0 | RND2_geneExp | 2.70E-05 | 0.00024448 | 0.00121655 |
| 0 | CLUHP3_geneExp | 2.66E-05 | 0.00026092 | 0.00121655 |
| 1 | GIMAP8_geneExp | 2.47E-05 | 0.00030982 | 0.00121655 |
| 1 | ADARB1_geneExp | 2.44E-05 | 0.00029688 | 0.00121655 |
| 1 | CAPN11_geneExp | 2.34E-05 | 0.00024773 | 0.00121655 |
| 0 | EPHA4_methylation | 2.34E-05 | 0.00024841 | 0.00121655 |
| 1 | GNA14_geneExp | 2.25E-05 | 0.00024502 | 0.00121655 |
| 1 | PRKRA_geneExp | 2.15E-05 | 0.00033126 | 0.00121655 |
| 1 | AACS_geneExp | 2.15E-05 | 0.00383796 | 0.00121655 |
| 0 | SLC48A1_geneExp | 2.13E-05 | 0.00036799 | 0.00121655 |
| 1 | MIS18A_geneExp | 2.09E-05 | 0.00024576 | 0.00121655 |
| 1 | DDIT3_geneExp | 2.03E-05 | 0.0002418 | 0.00121655 |
| 1 | ASNS_geneExp | 1.43E-05 | 0.00030792 | 0.00121655 |
| 0 | MST1L_geneExp | 1.41E-05 | 0.00024775 | 0.00121655 |
| 1 | CTNND1_methylation | 8.17E-06 | 0.0002944 | 0.00121655 |
| 1 | TMEM131L_geneExp | 3.86E-06 | 0.00053715 | 0.00121655 |
| 1 | ABCB6_geneExp | 1.08E-06 | 0.00371376 | 0.00121655 |

**Table S3** Enrichment analysis of Figure 3G

| #category | term ID |
| --- | --- |
| GO Function GO:0001605 Adrenomedullin receptor activity 3 3 2.29 0.0122 9606.ENSP00000242249 | 9606.ENSP00000253796 |
| GO Component GO:1903143 Adrenomedullin receptor complex 3 3 2.29 0.0051 9606.ENSP00000242249 | 9606.ENSP00000253796 |
| STRING clusters CL:6596 Mixed | incl. Mitotic Spindle Checkpoint |
| STRING clusters CL:6597 Mixed | incl. Amplification of signal from the kinetochores |
| STRING clusters CL:6604 Mixed | incl. Amplification of signal from the kinetochores |
| STRING clusters CL:6608 Mixed | incl. Regulation of mitotic sister chromatid segregation |
| STRING clusters CL:6610 Mixed | incl. Spindle elongation |
| STRING clusters CL:18723 Mixed | incl. Complement and coagulation cascades |
| STRING clusters CL:18724 Mixed | incl. Complement and coagulation cascades |
| STRING clusters CL:6614 Mixed | incl. Spindle elongation |
| Monarch EFO:0004747 Protein measurement 53 5856 0.25 0.0130 9606.ENSP00000013222 | 9606.ENSP00000052754 |
| TISSUES BTO:0000042 Animal 94 15148 0.09 0.0108 9606.ENSP00000013222 | 9606.ENSP00000052754 |
| COMPARTMENTS GOCC:1903143 Adrenomedullin receptor complex 3 5 2.07 0.0158 9606.ENSP00000242249 | 9606.ENSP00000253796 |
| COMPARTMENTS GOCC:1990406 CGRP receptor complex 3 5 2.07 0.0158 9606.ENSP00000242249 | 9606.ENSP00000253796 |
| UniProt Keywords KW-0131 Cell cycle 16 651 0.68 0.00015 9606.ENSP00000220514 | 9606.ENSP00000247191 |
